# The Effects of Shoe Sole Thickness on Running Biomechanics and Economy: A Systematic Review

**DOI:** 10.1186/s40798-026-01020-1

**Published:** 2026-04-29

**Authors:** Cagla Kettner, Felix Krapp, Thorsten Stein

**Affiliations:** BioMotion Center, Institute of Sports and Sports Science, Karlsruhe Institute of Technology, Karlsruhe, Germany

**Keywords:** Running footwear, Stack height, Kinematics, Kinetics, Running economy, Spatiotemporal variables, Advanced footwear technology

## Abstract

**Background:**

Advanced footwear technologies (AFT) are popular for their potential performance benefits, though concerns about injury risks persist. Among various AFT features, sole thickness is particularly debated, especially after World Athletics imposed a 40 mm limit to prevent unfair competitive advantages. However, the effects of sole thickness on running biomechanics and economy are not well understood, particularly because sole thickness often co-varies with other shoe characteristics in shoe designs.

**Objective:**

This review examines the effects of sole thickness on spatiotemporal variables, kinematics, kinetics, and running economy. The review focuses on studies in which sole thickness was the primary variable of interest.

**Methods:**

A systematic literature search was conducted following PRISMA guidelines. Eligible studies included original research on running with participants of all expertise levels, analyzing spatiotemporal variables, kinematics, kinetics, or running economy.

**Results:**

Fourteen studies met the criteria, mostly focusing on male recreational or experienced runners. Thicker soles were linked to increased stance time, while other spatiotemporal parameters remained unchanged. Significant effects were seen in ankle kinematics, with more dorsiflexion at initial contact with thicker soles, though knee and hip movements were less affected. Thicker soles increased peak eversion in the frontal plane. No consistent trends emerged for joint kinetics, stiffness, or center of mass movement. Vertical ground reaction force (GRF) peaks remained largely unchanged, but loading rates generally decreased with thicker soles. Only one study assessed running economy, with no significant effects. Overall, the certainty of evidence across outcomes was low to very low due to methodological heterogeneity and limited study numbers.

**Conclusions:**

Thicker soles were largely linked to longer stance times and lower GRF loading rates. Future research should comprehensively report shoe characteristics, include more diverse populations (e.g., female runners, forefoot strikers), and expand investigations to underexplored aspects such as muscle activity and movement coordination.

## Key Points

1. Thicker soles increase stance time and ankle dorsiflexion at initial contact but have largely inconsistent effects on other spatiotemporal parameters as well as joint kinematics.
2. Thicker soles reduce vertical ground reaction force loading rate, but their impact on stiffness and running economy is unclear.
3. Methodological heterogeneity and limited participant diversity highlight the need for more inclusive and comprehensive research.

## Background

Running shoes play a vital role in enhancing performance and reducing injury risk [1]. The complexity of modern running shoes, which incorporate multiple design elements, combined with the unique needs of different runner subgroups [1], makes determining the ideal shoe design challenging. Additional factors, such as habitual foot strike patterns [2] and running surface hardness [3], further complicate this process. Another challenge is the interaction of various design features, making it difficult to isolate their individual effects [4]. For example, incorporating a carbon plate in a shoe may require a thicker sole, increasing both the height and mass of the shoe. A systematic analysis of existing research is needed to evaluate the impact of specific shoe features on biomechanics and performance.

One key design feature of running shoes is sole thickness. In the footwear literature, the terms stack height, midsole thickness, and sole thickness are often used interchangeably to describe the vertical thickness of the shoe under the foot, although measurement locations and definitions may differ across studies [4–7]. In this review, the term sole thickness is used consistently, in line with World Athletics terminology [8], and refers to the vertical thickness of the shoe measured at the forefoot and heel unless otherwise specified. Since the 1970s running boom, shoe designs have substantially evolved [9]. Initially, cushioned shoes with thicker soles were developed to reduce vertical impact forces [9–11]. In the early 2000s, athletes at Stanford University trained barefoot, observing more natural landing mechanics [9]. This led to the creation of minimalist shoes designed to mimic barefoot running with a thin sole. Despite evidence that barefoot running and minimalist shoes can reduce impact forces [12] and alter spatiotemporal parameters [13, 14], no studies have demonstrated a reduction in injury risk with minimalist shoes [15–17]. Some research has shown increased risk of overuse injuries when transitioning to minimalist shoes [18, 19], although these injuries often seem to be linked to an abrupt transition rather than the shoes themselves.

In the 2010s, alongside minimalist shoes, maximalist footwear emerged with Advanced Footwear Technology (AFT) designed to enhance performance [20, 21]. The driver behind AFT development was the goal of breaking the two-hour marathon barrier, which was considered physiologically impossible by some experts and athletes [22–24]. AFT shoes feature technological advancements like carbon plates or rods to optimize shoe bending stiffness and minimize energy loss at the joints [25, 26]. These carbon elements are embedded in lightweight, thick midsole foam, providing cushioning and energy return [27]. Initially, World Athletics only regulated that the shoes must not offer unfair advantages and must be available to all athletes (Technical Rule 143.2, [28]). However, in 2020, World Athletics implemented rules limiting sole thickness to 40 mm and restricting rigid structures like carbon plates to a single element in the sole [29]. In 2022, regulations prohibited the use of intelligent technology in racing shoes [8].

AFT shoes are now widely used by runners of all skill levels [20, 30, 31]. Several studies have shown improvements in running economy with these shoes [20, 32, 33], though the benefits tend to decrease with lower skill levels [33, 34]. Concerns about potential injuries, particularly for non-elite runners, are growing [31]. While multiple studies have examined the biomechanical effects of AFT shoes, there is still no clear understanding of how individual shoe features influence performance and injury risk [4, 7, 35, 36]. Most research has compared different shoe models [27, 33, 37] rather than isolating specific design elements. A 2020 systematic review found limited studies on sole thickness, and their findings were inconclusive [38]. A more recent scoping review published in 2023 [10] analyzed various shoe features but did not focus on sole thickness. It found that while sole thickness influences vertical ground reaction forces (GRF), it does not affect foot and knee angles at initial contact (IC). Similarly, a narrative review on postmodern running shoes [7] included sole thickness as a sub-group of midsole geometry but did not explore the underlying biomechanical changes. Both reviews emphasized the need to understand how individual AFT features affect runners' responses and how runner-specific characteristics influence those responses.

While prior reviews have examined sole thickness as part of broader footwear features such as midsole materials and geometry [7, 10], a focused synthesis of studies systematically varying sole thickness is lacking. Accordingly, this systematic review provides a targeted review of studies examining how sole thickness impacts spatiotemporal variables, kinematics, kinetics, and running economy. Although this review targets studies in which sole thickness was explicitly manipulated, it is acknowledged that complete isolation from other footwear characteristics (e.g., heel-to-toe drop, midsole material properties, or mass) is rarely achievable in running shoe research.

## Methods

This systematic review was conducted and reported in accordance with the “Preferred Reporting Items for Systematic Reviews and Meta-Analyses (PRISMA 2020)” [39] and “Prisma in Exercise, Rehabilitation, Sport medicine and SporTs science (PERSiST)” Guidelines [40]. A review protocol was not prospectively registered; however, the eligibility criteria, search strategy, and analysis plan were defined a priori and applied consistently throughout the review process.

### Eligibility Criteria

Experimental studies using within-subject or repeated-measures designs were eligible for inclusion, while reviews were excluded, as in similar systematic reviews [41, 42]. Only full-length articles or short communications were considered, with conference proceedings excluded due to insufficient study details. Study eligibility was determined using the Population, Intervention, Comparison, and Outcome (PICO) framework [40, 43] as follows:

*Population *Healthy adult runners (aged 18–65 years) were included. No exclusion criteria were applied regarding foot strike pattern or running expertise level (e.g., non-elite, experienced runners).

*Intervention *Studies assessing different running shoe conditions during running were included. Other tasks such as walking were excluded. No restrictions were applied regarding the running surface (e.g., treadmill or overground), running duration, distance, or speed.

*Comparison *Studies were included if shoe sole thickness varied between conditions and was identified as the primary variable of interest in analyses of running performance, spatiotemporal characteristics, kinematics, or kinetics. Studies were not excluded if additional shoe features co-varied with sole thickness; however, barefoot conditions and comparisons in which sole thickness was not a defining factor were excluded.

*Outcome *Studies assessing running performance, spatiotemporal characteristics, kinematics, or kinetics were included.

### Data Sources and Search Strategy

A standardized electronic literature search was conducted using the following search term combinations: (“footwear” OR “shoes”) AND (“thickness” OR “stack height”) AND (“runn*” OR “jogg*”) AND (“biomechanic*” OR “econom*” OR “kinematic*” OR “spatio*” OR “kinetic*” OR “performance”). The literature search was conducted across three databases: Scopus, Web of Science, and PubMed. Additionally, a supplementary search was performed in *Footwear Science*, following the approach recommended in previous reviews on running shoes [42, 44]. The reference lists of relevant studies were also manually screened to identify any additional articles not captured through the database searches. No database filters related to study design or publication year were applied. Grey literature (e.g., conference abstracts, theses, and preprints) was not systematically searched due to the typically limited methodological detail available in these sources. Studies published up to 1 March 2025 were considered, and only articles published in English were included.

### Selection and Data Collection Process

One reviewer (FK) conducted the initial electronic search and removed duplicate records. Titles, abstracts, and full-text articles were independently screened for eligibility by two reviewers (CK and FK). Any disagreements were resolved by discussion, and if necessary, a third reviewer (TS) provided input to reach a consensus.

### Data Extraction

A data extraction form (Table 1) was developed based on previous systematic reviews in related areas and the PICO framework [38, 40, 41, 45]. The extracted data included: research question, study design, description of the sample, details of the tested shoes, experimental and analysis protocol, and the outcomes of the study. Data were extracted independently by two reviewers (CK and FK) and compared for potential disagreements.

**Table 1:** Data extraction items considered in this systematic review

Research question	Motivation/Aim
	Hypotheses
SampleSample	Number of participants and sex
	Power analysis
	Age, height, mass
	Experience level (e.g., in km/week or years)
	Footstrike pattern
	Ethics and written consent
	Withdrawals/dropouts
Shoes	Size
	Forefoot, midsole, heel thickness, heel-to-to drop
	Mass
	AFT (yes/no) – carbon elements in the sole
	Further shoe features
	Brand/Model or custom experiment shoes
Experiment design	Randomized/Parallelized order of the shoes
	Blinding
	Warm-up
	Familiarization to shoes
	Protocol (e.g., Duration, speed and number of runs)
	Fatigue control
	Overground/Treadmill
	Measurement devices
Analysis	Software and models used
	Filtering
	Step detection method
	Leg side
	Number of steps
	Analyzed parameters
	Statistical tests
	Effect sizes reported (Yes/No)
Outcomes	Spatiotemporal variables
	Kinematics
	Kinetics
	Running economy

*AFT* Advanced footwear technology

### Study Risk of Bias Assessment

The risk of bias for each included study was independently assessed by two reviewers (CK and FK) using the Cochrane risk of bias instrument tool (RoB-2) [46], following the approach of similar running-related studies [47]. Although RoB-2 was originally developed for randomized clinical trials, no validated risk-of-bias tool currently exists for acute, within-subject biomechanical experiments. Therefore, RoB-2 was applied as a structured framework to assess internal validity, rather than as a clinical trial risk-of-bias assessment.

The RoB-2 crossover-trial framework was used to account for the within-subject nature of footwear comparisons. Within the domain *bias arising from the randomization process*, the randomization or counterbalancing of shoe allocation and the reporting of a priori power calculations were examined. *Bias due to deviations from intended interventions* was evaluated by considering protocol compliance, fatigue control, and the presence or absence of shoe familiarization periods. *Bias in measurement of the outcome* was assessed by determining whether outcome assessors were blinded to shoe condition and whether validated measurement devices and established biomechanical models were employed. *Bias in selection of the reported results* was evaluated based on the reporting of effect sizes and the use of appropriate corrections for multiple post-hoc comparisons.

Potential design-related sources of uncertainty, such as habitual foot-strike pattern, variation in shoe mass or heel-to-toe drop, and participant expertise level, were not treated as a separate risk-of-bias domain, but are discussed qualitatively in the Results and Discussion sections as factors that may influence the interpretation of findings. Risk-of-bias assessments were conducted independently by CK and FK. In cases of disagreement, consensus was achieved through discussion.

### Data Synthesis and Analysis

A meta-analysis was not feasible due to limited reporting of effect sizes and variance measures, inconsistent outcome variables, heterogeneous experimental protocols, and insufficient information to calculate standardized effects for repeated-measures designs across the included studies.

### Certainty of Evidence

Due to substantial heterogeneity in footwear interventions, study designs, and biomechanical outcome measures, a formal GRADE assessment was not feasible [40]. Instead, a qualitative certainty-of-evidence assessment was conducted within this systematic review using a narrative synthesis approach, which is recommended when quantitative synthesis is not appropriate. Certainty was judged for each main outcome domain by considering study design, sample size, risk of bias, consistency of findings, and the presence of potential confounding footwear characteristics, consistent with guidance for structured narrative synthesis in systematic reviews [48], and informed by principles outlined in the Cochrane Handbook for Systematic Reviews of Interventions and GRADE methodology literature [49, 50].

## Results

### Search Results and Study Selection

**Fig. 1:**
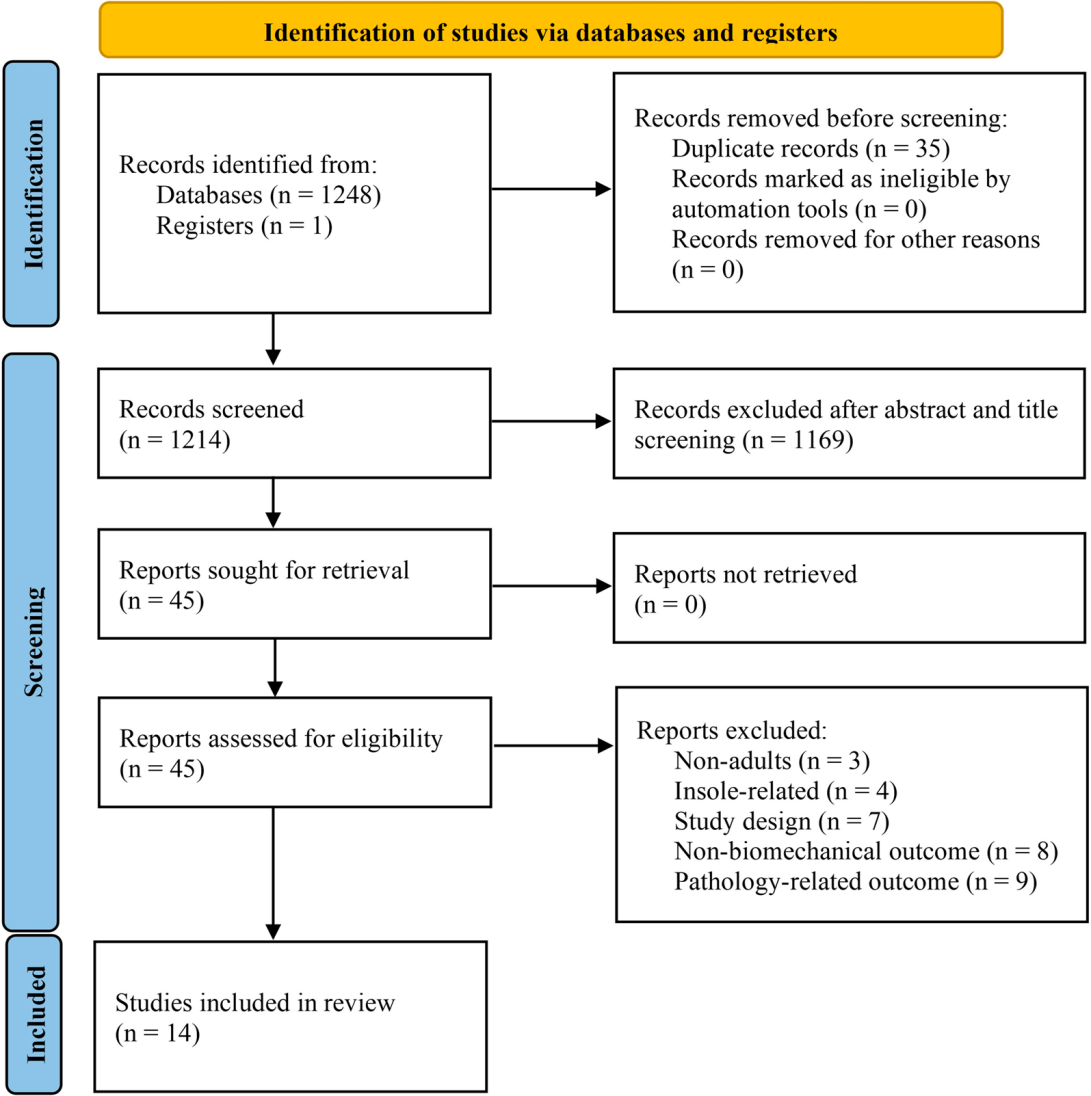
Flow diagram of the systematic search. Adapted from PRISMA 2020 flow diagram template for systematic reviews [39]

The initial search yielded 1249 articles, which were reduced to 1214 after removing duplicates (Fig. 1). Of these, 45 articles were assessed for eligibility, and 14 studies met the inclusion criteria, all employing experimental within-subject designs. Eleven studies explicitly mentioned sole thickness in their title or research question [51–61], while one study focused on midsole geometry, including both heel-to-toe drop and sole thickness [62]. One study compared minimal versus nontraditional shoes [63] and another compared maximal versus traditional shoes [64], primarily differing in sole thickness.

### Risk of Bias in Studies

Table 2 presents the estimated risk of bias for each study, with an overall judgment ranging from low risk to some concerns, based on the RoB-2 tool. The primary sources of concern were identified as the absence of power analyses and effect sizes, potential carry-over effects due to missing shoe familiarization sessions, and the lack of shoe blinding. Confounding factors, specifically habitual foot strike patterns and unreported heel-to-toe drop and/or shoe mass, were also repeatedly detected as sources of potential bias.

**Table 2:**
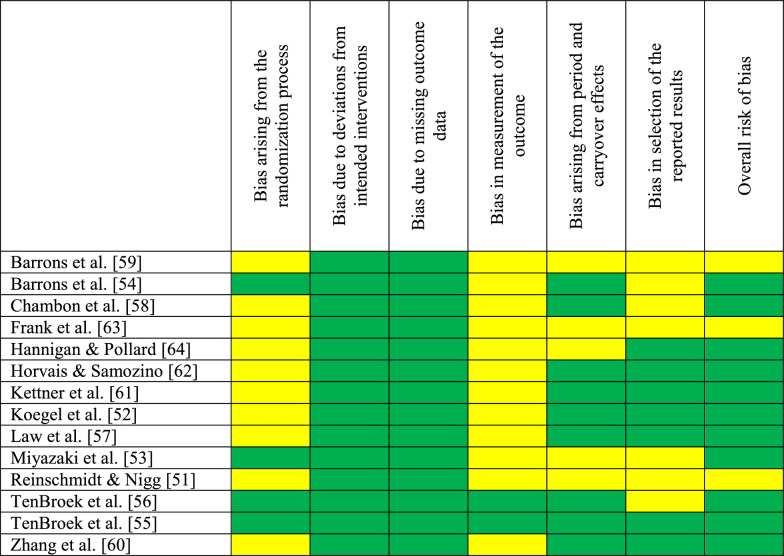
Estimated risk of bias of the reviewed studies based on the Cochrane risk of bias instrument tool (RoB-2) [1]

*Green* Low risk, *Yellow* Some concerns, *Red* High risk

Risk-of-bias judgments were used to inform the narrative synthesis and certainty-of-evidence assessment and were not used to weight outcomes quantitatively

### Certainty of Evidence

Overall, the certainty of evidence across outcome domains was judged to be low to very low. Evidence for spatiotemporal variables and ankle kinematics demonstrated relatively consistent directional trends across studies but was limited by small sample sizes, lack of a priori power calculations, short-term laboratory designs, and potential confounding footwear characteristics, resulting in low certainty. Evidence for joint kinetics, stiffness measures, center of mass (COM) movement, and running economy was inconsistent across studies and based on few investigations, leading to very low certainty.

### Sample Size and Characteristics

An overview of the sample size and characteristics is presented in Table 3. Four studies performed a priori power calculations [53–56]. The sample size ranged from 5 to 31 participants, with a mean of 15.4 ± 6.7 across the 14 studies, totaling 216 participants (87% male, 13% female). Mean age was 27.8 ± 6.7 years, ranging from 20 to 35.8 years. Participant height (mean 1.76 ± 0.05 m) and mass (68.9 ± 6.8 kg) were reported in most studies.

**Table 3:** Sample size and characteristics of reviewed studies

	Sample	Power calculation	Age	Height	Weight	Experience	Footstrike
	#	Yes/no	Years	cm	kg	−	−
Barrons et al. [59]	21 (10F)	No	24.3 ± 4.4	1.75 ± 0.07	69.9 ± 8.8	Recreational (≥ 19 km/week)	−
Barrons et al. [54]	13 (14F)	Yes	−	1.73 ± 0.05	66.9 ± 5.3	Recreational	−
Chambon et al. [58]	15	No	23.9 ± 3.2	1.77 ± 0.03	73 ± 8	Physically active	Rearfoot
Frank et al. [63]	24 [N:12, T:12]	No	N:21.5 ± 2.7 T:23.5 ± 5.8	N:1.77 ± 0.06 T:1.75 ± 0.05	N:75.8 ± 9.8 T: 68.3 ± 5.1	N: < 10 km last year; T: ≥ 30 km/week (61.7 ± 28.2 km/week; easy pace: 4:37 ± 0:23 min/km, hard pace: 3:36 ± 0:20 min/km)	−
Hannigan and Pollard [64]	20 (14F)	No	32.3 ± 6.1	1.69 ± 0.08	65.5 ± 10.1	≥ 16 km/week (24.5 ± 10.6 km/week, running for 10.3 ± 7.2 years)	Rearfoot
Horvais and Samozino [62]	12	No	35.8 ± 12.9	1.76 ± 0.05	69.3 ± 5.4	Regular (≥ 20 km/week)	Rearfoot
Kettner et al. [61]	17	No	25.7 ± 3.9	1.77 ± 0.04	68.1 ± 6.0	Experienced (33.7 ± 22.4 km/week, 4.2 ± 1.8 days/week)	−
Koegel et al. [52]	31	No	31.1 ± 6.9	1.81 ± 0.06	72.5 ± 6.0	10 km in ≤ 44 min (35.46 ± 2.89 min, n = 24) and/or 5 km in ≤ 22 min (16.66 ± 1.67 min, n = 25)	−
Law et al. [57]	15	No	31.4 ± 13.2	1.73 ± 0.03	64.8 ± 5.4	Recreational (≥ 12 km/week)	Rearfoot
Miyazaki et al. [53]	14	Yes	20 ± 1	1.74 ± 0.05	58.3 ± 4.0	Running for ≥ 2 years	Non-rearfoot
Reinschmidt and Nigg [51]	5	No	31.6 ± 6.8	−	72.7 ± 6.2	Physically active	−
TenBroek et al. [56]	10	Yes	−	−	−	Recreational	Rearfoot
TenBroek et al. [55]	10	Yes	−	−	−	Recreational, comfortable running for 30 min without serious fatigue	Rearfoot
Zhang et al. [60]	12	No	26.9 ± 11.0	1.81 ± 0.05	73.6 ± 8.3	Recreational (28.7 ± 18.3 km/week)	Rearfoot

*N* Novice,* T* Trained

Most studies predominantly included male recreational runners. Number of female (F) participants are indicated in parentheses if the study included F participants

Expertise level definitions varied: six studies classified participants as "recreational runners" [54–57, 59, 60], with two specifying weekly mileage thresholds, while others described participants as "physically active" [51, 58], “regular” [62] or “experienced” [61] based on mileage or running history. Eight studies controlled footstrike pattern [55–58, 60, 62, 64], with seven including only rearfoot strikers and one including non-rearfoot strikers [53].

All included studies except one [51] reported approval by an institutional ethics committee; however, informed consent was obtained from all participants prior to testing in all studies. No study reported adverse events related to the experimental procedures. Participant dropouts or withdrawals were not reported, and no analyses were described as being affected by missing data.

### Running Shoe Characteristics

**Fig. 2:**
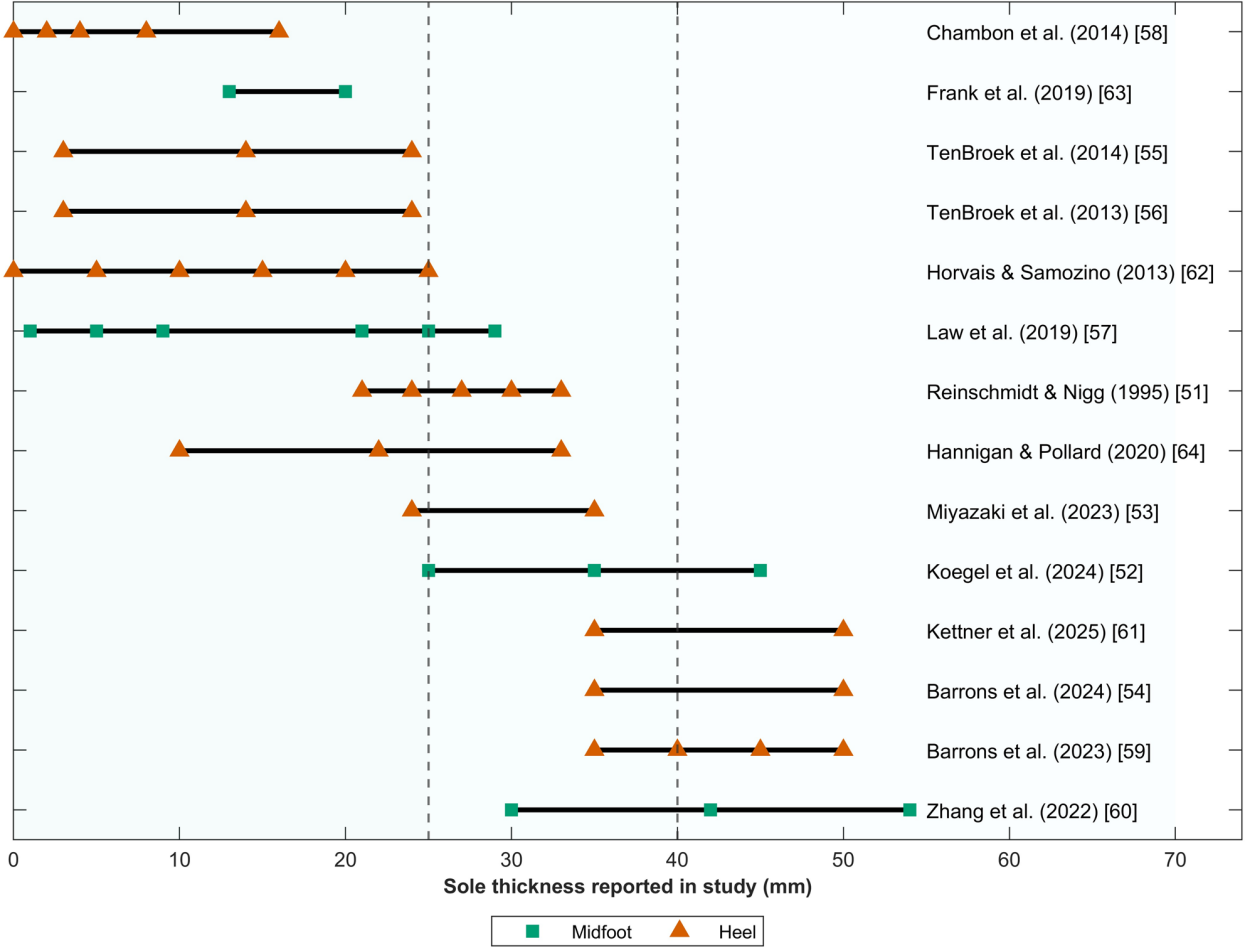
Overview of sole thickness ranges investigated across included studies. Horizontal bars represent the minimum and maximum sole thicknesses reported per study. Filled symbols indicate reported measurement locations (midfoot or heel). Vertical dashed reference lines indicate the three sole thickness categories used in this review: minimal to traditional footwear (0–25 mm), traditional to moderately thick soles (25–40 mm), and moderately thick to exaggerated soles (> 40 mm)

An overview of the characteristics of the tested running shoes is presented in Table 4. The studies compared between two and 16 shoes, with 12 using experiment-specific shoes [52, 54–56, 58–65]. Sole thickness was measured at the midsole [52, 57, 60], heel [51, 59], or both [53–56, 58, 61–64].To facilitate comparison across studies, sole thickness ranges were grouped pragmatically into minimal-to-traditional (0–25 mm), traditional-to-moderately thick (25–40 mm), and moderately thick-to-exaggerated categories (> 40 mm). These groupings reflect commonly studied footwear ranges and the World Athletics stack-height regulation [8], and are intended to describe the distribution of the existing evidence rather than to define physiological thresholds (Fig. 2).

**Table 4:** Characteristics of the running shoes used in the reviewed studies

	Size	Thickness			Heel-to-toe drop	Mass	Carbon plate/ rod	Bending stiffness	Energy return	Cushioning (forefoot-heel)	Midsole hardness	Midsole material	Brand/model
		Forefoot	Midfoot	Heel									
	US	mm	mm	mm	mm	g		Nm/°	%	N/mm			
Barrons et al. [59]	9	−		35, 40, 45, 50	All 7	214.5, 229.5, 241.5, 260.5 (before normalization)	Yes to all	0.187, 0.171, 0.160, 0.166	83.5, 84, 85, 86.5	201–176, 172–155, 151–125, 131–100	−	TPEE	Experiment shoes, provided by Adidas
Barrons et al. [54]	9	28, 43		35, 50	(All 7)	214.5, 260.5	Yes to all	0.187, 0.166	83.5, 86.5	201/176, 131/100	−	TPEE	Experiment shoes, provided by Adidas
Chambon et al. [58]	9	BF^a^, 0, 2, 4, 8, 16		BF^a^, 0, 2, 4, 8, 16	All 0	−	−	−	−	−	60 Asker C	EVA	−
Frank et al. [63]	−		13, 13, 20, 20	−	4, 4, 12, 12	−	−	−	−	−	40 Asker C (Soft) and 70 Asker C (Hard), two shoes each	−	Experiment shoes, with Nike logo
Hannigan and Pollard [64]	8/ 10.5	6, 18, 29	−	10, 22, 33	All 4	−	−	−	−	−	−	Fresh Foam (New Balance)	Experiment shoes, provided by New Balance
Horvais and Samozino [62]	−	16 shoes differing btw. 0–25		16 shoes differing btw. 0–25	16 shoes differing btw. 0–15	−	−	−	−	−	55 Asker C	−	Experiment shoes, with Salomon logo
Kettner et al. [61]	9	19^a^, 28, 43	−	27^a^, 35, 50	8^a^, 7, 7	219^a^, 220, 268	No^a^, Yes, Yes	−	−	−	−	Identical	Experiment shoes, provided by Adidas
Koegel et al. [52]	9/ 11	−	25, 35, 45	−	−	186/229, 211/229, 202/246	Yes to all	0.22/0.22, 020/0.23, 0.20/0.21	83/80, 82/82, 83/83	136–128/155–144, 100–86/108–99, 77–66/83–76	−	TPEE	Experiment shoes, provided by Adidas
Law et al. [57]	9	−	1, 5, 9, 21, 25, 29	−	All 0	−	−	−	−	−	50 Asker-C	EVA	Experiment shoes
Miyazaki et al. [53]	8.5	5^a^, 18, 25	−	5^a^, 24, 35	(0, 6, 10)	229^a^, 217, 228	−	−	−	−	−	Fresh Foam (New Balance)	Xero Prio^a^, New Balance Tempo, New Balance Altom
Reinschmidt and Nigg [51]	9	−	−	21, 24, 27, 30, 33	5.0, 6.1, 7.2, 8.3, 9.5 (in °)	−	−	−	−	−	Shore A 60	−	−
TenBroek et al. [56]	−	BF^a^, 3, 9, 12	−	BF^a^, 3, 14, 24	BF^a^, 0, 5, 12	N/A^a^, 163.9, 199.9, 236.7	−	−	−	−	61 Asker C	EVA	Experiment shoes, with New Balance logo
TenBroek et al. [55]	−	BF^a^, 3, 9, 12	−	BF^a^, 3, 14, 24	BF^a^, 0, 5, 12	N/A^a^, 163.9, 199.9, 236.7	−	−	−	−	61 Asker C	EVA	Experiment shoes, with New Balance logo
Zhang et al. [60]	10	−	30, 42, 54	−	7, 5, 3	221, 233, 268	−	−	−	−	−	identical	Experiment shoes, provided by New Balance

*BF* Barefoot, *N/A* Not applicable, *TPEE* Thermoplastic polyester elastomer, *EVA* Ethylene–vinyl acetate

Shoe size was converted to United States sizing (US) if other units were used in the studies

Superscript ^a^ indicates the shoes that were not considered in this systematic review since they did not meet inclusion criteria

The heel-to-drop values in parentheses are not reported in the reviewed studies but calculated based on forefoot and heel height

The heel-to-toe drop was reported in most studies, varying between 0 and 12 mm. Eight studies reported shoe mass [52–56, 59, 60], with two normalizing masses by attaching weights on shoes [59, 60]. Most studies (n = 10) reported shoe material characteristics, such as hardness [51, 55–58, 62, 63] or bending stiffness energy return, and cushioning [52, 54, 59]. Four studies tested carbon-infused soles [52, 54, 59, 61].

### Study Design

Study designs are summarized in Table 5. All but one study consisted of a single measurement session; this study [55] had three sessions on different days.

**Table 5:** Study design of the reviewed studies

	Warm-up	Warm-up shoes	Shoe familiarization	Protocol per shoe	Shoe order	Running environment	Fatigue control/breaks
Barrons et al. [59]	Treadmill. incremental test (2.4 m/s then + 0.2 m/s) to determine anareobic threshold speed before measurements. Value of speeds not reported	Adidas Alphabounce	−	6 min. Last 2 min measured	Randomized	Treadmill (Laboratory)	10 min break between shoes
Barrons et al. [54]	No extra	Shoe of each condition	Overground. Five warm-up trials for each new shoe-turn combination	Curved runway with three radii conditions (3 m, 6 m and 9 m). Ten valid trials at 4–5 m/s	Randomized	Overground (Laboratory)	−
Chambon et al. [58]	No extra	Shoe of each condition	Treadmill. Run at 3.3 m/s for 3 min with each new shoe condition	15 m runway (force plate is located at 10 m). 5 valid trials at 3.3 m/s (± 5%)	Randomized	Overground (Laboratory)	−
Frank et al. [63]	Treadmill. Speed increased until a RPE score equal to ‘3’ or a moderate effort	−	−	4 min at self-selected running speed which was different between groups while the relative intensity was normalized (3.1 ± 0.4 m/s trained; 2.4 ± 0.2 m/s novices)	Randomized	Treadmill (Laboratory)	Enough break
Hannigan and Pollard [64]	Treadmill. 5 min easy pace. Overground: three to five practice trials	−	−	10–15 m runway. 5 valid trials at self-selected speed (males: 2.8 ± 0.1 m/s, females: 2.9 ± 0.1 m/s)	Randomized	Overground (Laboratory)	−
Horvais and Samozino [62]	Treadmill. 10 min at self-selected speed	Own shoes	Last 20 s collected	1 min at 3.9 and 4.7 m/s. Last 20 s collected	Randomized	Treadmill (Laboratory)	2 min break between shoes
Kettner et al. [61]	Treadmill. 5 min self-selected speed	Own shoes	Treadmill. Run at self-selected speed for 3 min and walk at 5 km/h with new shoe conditions	1.5 min at 2.8 and 4.2 m/s	Parallelized	Treadmill (Laboratory)	2 min break between speed, 5 min between shoes. Borg scale used
Koegel et al. [52]	Overground. 8 min warm-up self-selected speed	Own shoes	Overground. First 500 m cut in data	1.2 km at individual 10 km race pace	Randomized	Overground (Field)	5 min between shoes
Law et al. [57]	Treadmill. 5 min warm-up self-selected speed	Own shoes	Overground. First 2:40 min run at self-selected speed as familiarization	3 min at self-selected speed (last 20 s recorded)	Randomized	Treadmill (Laboratory)	Break between shoes if necessary
Miyazaki et al. [53]	Treadmill. 5 min + Overground: 5 min at self-selected	Own shoes	−	20 m runway. 5 trials at 4.2 m/s ± 5%	Parallelized and blinded	Overground (Laboratory)	≥ 5 min
Reinschmidt and Nigg [51]	No warm-up but familiarization to protocol	−	−	10 m runway. 5 valid trials at 4.6 ± 0.2 m/s	Parallelized and blinded	Overground (Laboratory)	−
TenBroek et al. [56]	Treadmill. Standardized warmup	Own shoes	No prior shoe familiarization (intentional)	6 min at 3 m/s	Parallelized	Treadmill (Laboratory)	Speed chosen to avoid fatigue, enough breaks
TenBroek et al. [55]	Treadmill. Standardized warmup	Own shoes	No prior shoe familiarization (intentional)	Laboratory. Treadmill. 30 min at 3 m/s. Shoes parallelized			Three measurement days with at least one day off
Zhang et al. [60]	No extra	Shoe of each condition	At least 5 min	Laboratory. Overground. 40 m runway. 5 valid trials at 4.5 m/s ± 5%. Shoes parallelized			Break allowed

*RPE* Rating of perceived exertion

Speed as converted to m/s if it was given in km/h in the reviewed study

#### Warm-up and Shoe Familiarization

All studies included either a warm-up or a shoe familiarization session, with some using both [52, 57, 61]. Warm-up durations ranged from 5 to 10 min [52, 53, 57, 61, 62, 64], with running speeds either self-selected [52, 53, 57, 61, 62] or at an easy pace [64]. Shoe familiarization durations varied (e.g., 2:40–5 min, or 5 trials). One study used standardized warm-up shoes [59], while seven studies stated that the participants wore their own shoes during warm-up [52, 53, 55–57, 61, 62].

#### Running Protocol

Measurements were taken either on a treadmill [55–57, 59, 61–63] or during overground running [51–54, 58, 60, 64], with one overground study taking place outdoors on concrete [52]. Treadmill durations ranged from 1 to 30 min [55–57, 59, 61–63], and overground runs were typically 10–40 m, with 5 valid trials recorded [51, 53, 58, 60, 64]. Running speeds ranged from 2.4 to 5 m/s [51, 53–56, 58, 60–63], although four studies did not report speed [52, 57, 59, 64]. All studies used randomized or parallelized orders, and two used blinded designs [55, 56]. Most studies allowed breaks between conditions, ranging from 2 to 10 min [52, 53, 59, 61, 62] or based on participant needs [56, 57, 60, 63].

### Data Recording and Analysis

Data recording and analysis methods are detailed in Table 6. Most studies (n = 12) used motion capturing with infrared cameras for kinematic data [51, 53–61, 63, 64], while two also used accelerometers [55, 56]. Timing gates were used to control running speed in six studies [51, 53, 54, 58, 60, 64]. Force measurements were taken in eight studies using force plates [51, 53, 54, 58, 60, 64] or an instrumented treadmill [57, 59]. Most studies (n = 11) used a Butterworth low-pass filter (2nd or 4th order, 8–50 Hz). Step detection was based on a threshold for force data [53, 54, 57, 59, 60] or kinematics [55, 56, 61], while six studies did not report step detection methods [51, 52, 58, 62–64].

**Table 6:** Data recording and analysis of the reviewed studies

	Measurement devices	Softwares and modeling	Data filtering	Step detection	Right/left leg	Number of steps	Kinematics/kinetics model	Statistical tests	Effect sizes
Barrons et al. [59]	MoCap, FP in treadmill	Visual 3D, MATLAB, SPSS	Butterworth, 4th order, low-pass, 15-Hz (marker and force)	F_t_ = 20 N	Left	−	Lower body	rmANOVA, post-hoc tests with Bonferroni corrections	No
Barrons et al. [54]	MoCap, FP, timing gates	Visual 3D, MATLAB, SPSS	Butterworth, 4th order, low-pass, 15-Hz (marker and force)	F_t_ = 20 N	Right (outer foot)	10 trials	Lower body	rmANOVA, post-hoc tests with Bonferroni corrections	No
Chambon et al. [58]	MoCap, FP, timing gates, high-speed camera, accelerometer	Nexus, OpenSim (generic model), Statistica	Butterworth, 2nd order, low pass, 50 Hz (force and acceleration)	−	Right	5 trials	Lower body	rmANOVA, post-hoc Tukey tests	No
Frank et al. [63]	MoCap, heart rate monitor	Visual 3D	Butterworth, 2nd order, low pass, 15 Hz (marker)	−	Right	216 steps	Lower body	Mixed general linear model, post-hoc paired t-tests	No
Hannigan and Pollard [64]	MoCap, FP, timing gates, video camera	Nexus, C Motion, Excel, SPSS, G-power	Butterworth, 4th order, low pass, 12 Hz (marker), 50 Hz (force)	−	Dominant leg	4 trials	Lower body	rmANOVA, post-hoc tests with Bonferoni corrections	Yes
Horvais and Samozino [62]	Optical measurement system, high-speed camera	−	−	Cameras (OptoJump)	−	30–40 steps	N/A	Spearman correlations	N/A
Kettner et al. [61]	MoCap	Nexus, OpenSim (modified Hamner running model), MATLAB, SPSS	Butterworth, 4th order, low pass, 10 Hz (marker)	Kinematic-based	Left	20 steps (linear analysis), 100 steps (nonlinear analysis)	Full body	rmANOVA, post-hoc paired t-tests with Bonferroni Holm corrections	Yes
Koegel et al. [52]	Commercial wearable sensor on shoe and running watch	−	−	Kinematic-based	Right	−	N/A	PCA, and clustering with Ward's method	N/A
Law et al. [57]	MoCap, FP in treadmill	−	Butterworth, 4th order, low pass, 8 Hz (marker), 50 Hz (force)	F_t_ = 10 N	−	20 steps	Ankle	rmANOVA, post-hoc t-tests Bonferroni corrections	Yes
Miyazaki et al. [53]	MoCap, FP, timing gates	Cortex, MATLAB, SPSS	Butterworth, 4th order low pass, 12 Hz (marker), force data filtering not stated	F_t_ = 50 N	Right	5 trials	Full body	Friedman tests, post-hoc tests with Bonferroni Holm corrections	No
Reinschmidt and Nigg [51]	MoCap, FP, timing gates	Kintrak	Butterworth, 4th order, low pass, 16 Hz (marker & force)	−	Right	5 trials	Ankle	rmANOVA	No
TenBroek et al. [56]	MoCap, accelerometer	Visual 3D	Butterworth, 2nd order, low pass, 12 Hz (marker); 50 Hz (accelerometer)	Kinematic-based	Left	10 steps	Lower body	rmANOVA, post-hoc Tukey tests	No
TenBroek et al. [55]	MoCap, accelerometer	−	Butterworth, 2nd order, low pass, 12 Hz (marker data); 50 Hz (accelerometer)	Kinematic-based	Left	10 steps	Lower body	rmANOVA, post-hoc Tukey tests	Yes

*MoCap* Motion capturing with infrared cameras, *FP* Force plates. *F*_*t*_ Force threshold, *rmANOVA* Repeated measures analysis of variance, *PCA* Principal component analysis, *N/A* Not applicable

The spatiotemporal, kinematic, and kinetic parameters analyzed varied across studies (Table 7). Stance time was the most frequently analyzed parameter (n = 8, [52, 53, 55–59, 62]), followed by step/stride frequency (n = 5, [52, 57, 59, 61, 62] and stride length (n = 3, [52, 57, 59]). Duty factor [61, 62] and flight time [52, 62] were each analyzed in two studies, and detrended fluctuation of stride time [61], ratio of braking to propulsion duration [61], time to complete the turn task [54], and running speed [52] were analyzed in one study each.

**Table 7:** Overview of analyzed parameters and reported results of the reviewed studies

	Sole thickness contrast (mm)	Domain	Analyzed parameters	Results
Barrons et al. [59]	35, 40, 45, 50	Spatiotemporal	Stance time	−
			Stride frequency	−
			Stride length	−
		Kinematics	Ankle, hip, knee sagittal peak angles during stance, at IC and TO	Peak dorsiflexion S50 < S35 and S50 < S40
			Peak ankle eversion	S45 > S35
			Leg length	At IC: S50 > S35, S50 > S40, S45 > S35; at mid-stance: greater sole thickness led to a longer effective leg, with all pairwise comparisons significant, except S40 vs. S45; at TO: S50 > all lower soles, and S45 > S35
		Running economy	Running economy (average VO_2_ and energetic cost)	
Barrons et al. [54]	35, 50	Spatiotemporal	Time to complete task	−
		Kinematics	Peak frontal ankle angle	−
			Velocity of COM	−
		Kinetics	Resultant, vertical, medial, braking, and propulsive peak of GRF	Peak propulsive GRF S50 < S35 in running turn task
Chambon et al. [58]	0, 2, 4, 8, 16	Spatiotemporal	Stance time	S16 > S0
		Kinematics	Strike index and foot strike angle at IC	−
			Ankle, knee and hip flexion angle at IC	−
			Ankle, knee and hip flexion ROM during stance	−
			Tibial acceleration peak and rate	−
		Kinetics	Maximal ankle, knee and hip flexion moment during stance	−
			Average loading rate and transient peak of vGRF	−
Frank et al. [63]	13, 20^a^	Kinematics	Dynamic stability of sagittal joint angles	−
Hannigan andPollard [64]	10, 22, 33	Kinematics	Dorsiflexion (peak and ROM during stance, at IC and TO)	Dorsiflexion S22 > S10; ROM S33 > S10 and S22 > S10
			Ankle eversion (peak and ROM during stance, at IC and TO, duration%)	Peak ankle eversion S33 > S22; eversion duration S33 > S22 and S33 > S10
		GRF and loading	Transient peak, loading rate, and vertical active peak of GRF	Vertical average loading rate S33 < S10
Horvais and Samozino [62]	0, 5, 10, 15, 20	Spatiotemporal	Stance time	−
			Flight time	−
			Step frequency	−
			Duty factor	−
		Kinematics	Foot angle at IC,	Thicker soles were associated with a stronger rearfoot strike pattern
		Stiffness	Vertical and leg stiffness	Greater heel thickness was associated with lower leg stiffness at 4.7 m/s
Kettner et al. [61]	35, 50	Spatiotemporal	Step frequency (leg-length normalized)	S50 > S35 at 4.2 m/s
			Duty factor	−
		Kinematics	Time series of sagittal ankle, knee and hip angles	Ankle differed between shoes with no post-hoc differences
			Time series of frontal ankle, knee and hip angles	Ankle inversion S35 > S50
			Foot eversion and inversion in frontal plane	Peak eversion S50 > S35
			Vertical COM oscillation	S50 > S35
			Dynamic stability of body segments	−
		Vertical/leg stiffness	Vertical and leg stiffness	−
Koegel et al. [52]	25, 35, 45^a^	Spatiotemporal	Stance time	Third most influential factor in clustering runners
			Flight time	Fourth most influential factor in clustering runners
			Step frequency	Small contribution to cluster formation
			Stride length	Small contribution to cluster formation
			Running speed	No significant differences between clusters
		Kinematics	Vertical COM oscillation	Second most influential factor in clustering runners
		Vertical/Leg stiffness	Leg stiffness	Most influential factor in clustering runners
Law et al. [57]	1, 5, 9, 21, 25, 29	Spatiotemporal	Stance time	S9^a^ > S1^a^, S21^a^ > S^a^, S25^a^ > S1^a^, S29^a^ > S1^a^, and S25^a^ > S5^a^
			Step frequency	−
			Stride length	−
		Kinematics	Foot strike angle	S25^a^ > S1^a^
		GRF and loading	Average and instantaneous loading rate of vGRF	Average loading rate: S25^a^ < S1^a^, S25^a^ < S5^a^, S21^a^ < S1^a^, S9^a^ < S1^a^, S9^a^ < S5^a^ and S5^a^ < S1^a^; instantaneous loading rate: S29^a^ < S1^a^, S25^a^ < S1^a^, S25^a^ < S5^a^, S21^a^ < S1^a^, S21^a^ < S5^a^, S9^a^ < S1^a^ and S9^a^ < S5^a^
Miyazaki et al. [53]	24, 35	Spatiotemporal	Stance time	S35 > S24
		Kinematics	Footstrike angle	−
			Ankle and knee sagittal peak angle	Peak dorsiflexion S35 > S24
		Kinetics	Ankle and knee sagittal moment and work	Positive ankle work for S35 > S24, negative ankle work for S35 > S24
Reinschmidt and Nigg [51]	21, 24, 27, 30, 33	Kinetics	Min and max of flexion/extention moment of ankle during stance and its occurrence in %	Max moment increased by 6.3 Nm for every 10 mm increase in heel thickness
TenBroek et al. [56]	3, 14, 24	Spatiotemporal	Stance time	S24 > S3 and S14 > S3
		Kinematics	Sagittal ankle and knee angle at IC	Dorsiflexion S14 > S3 and S24 > S3, knee flexion S14 > S24
			Sagittal foot, leg and thigh angle at IC	Foot angle S24 > S3 and S14 > S3, leg angle S24 > S3, thigh angle S14 > S3 and S24 < S14
			Knee flexion ROM	S24 > S3, S14 > S3
			Frontal ankle angle at IC	−
			Ankle eversion ROM	S24 < S3, S14 < S3
			Tibial internal rotation ROM	S24 < S3
			Peak tibialis and head accelerations and transfer function	Tibialis: S24 < S14 < S3, head: S24 < S23 and S24 < S14
TenBroek et al. [55]	3, 13, 24	Spatiotemporal	Stance time	S24 > S3 and S24 > S14
		Kinematics	Sagittal ankle and knee angle at IC	Dorsiflexion S24 > S3 and S24 > S14, knee flexion S14 > S3 and S24 > S3
			Sagittal foot, leg and thigh angle at IC	Foot dorsiflexion S24 > S3 and S24 > S14, thigh flexion S14 > S3 and S24 > S3
			Peak knee flexion	S24 > S14
			Knee flexion ROM	S24 > S3 and S24 > S14
			Frontal ankle angle at IC	−
			Frontal foot and leg angle at IC	−
			Peak foot eversion	Peak S24 < S3 and S14 < S3
			Ankle eversion ROM	S14 > S24
			Peak thigh internal rotation and ROM	Rotation S24 < S3 and S24 < S14
			Peak tibial internal rotation and ROM	Rotation S24 < S3, ROM S24 < S3, S14 < S3
			Peak tibialis and head accelerations and transfer function	Tibialis: S3 > S24, head: S3 > S14 and S3 > S24
Zhang et al. [60]	30, 42, 54^a^	Kinematics	Shank retraction angle and time prior to IC	−
			Shank retraction angular velocity, horizontal and vertical heel velocity at IC	Shank retraction velocity S54^a^ < S30^a^ and S54^a^ < S42^a^, horizontal heel velocity S54^a^ > S30^a^
			Foot strike angle and shank angle at IC	Foot strike angle S54^a^ < S30^a^ and S42^a^ < S30^a^
			Peak ankle dorsiflexion and knee flexion velocity	Peak ankle dorsiflexion velocity S54^a^ < S42^a^ < S30^a^
			Ankle and knee ROM	−
		GRF and loading	Average and instantaneous loading rate of vGRF	Both S54^a^ > S42^a^ and S54^a^ > S30^a^
		Vertical/Leg stiffness	Ankle and knee stiffness	Ankle: S54^a^ > S42^a^ > S30^a^, knee: S54^a^ > S30^a^ and S54^a^ > S42^a^

*IC* Initial contact, *TO* Toe-off, *ROM* Range of motion, *COM* Center of mass, *rmANOVA* Repeated measures analysis of variance, *GRF* Ground reaction force, *vGRF* Vertical ground reaction force, *VO₂* Oxygen consumption

Only significant results are stated

The symbol – indicates no significant shoe effects

Standardized effect sizes are not shown due to limited reporting and methodological heterogeneity

The symbols “ < ” and “ > ” indicate the direction of change in the analyzed parameters between shoe conditions.

Shoe abbreviations include "S" followed by the heel thickness in millimeters (e.g., S3 for shoes with a 3 mm heel thickness)

If heel thickness was not reported, midsole thickness is used instead and marked with a subscript ^a^ (e.g., S25 ^a^ for shoes with a 25 mm midsole thickness)

For kinematics calculations, eight studies used a lower body model [54–56, 58–60, 63, 64], while two studies used an ankle model [51, 57], and another two employed a full body model [53, 61]. The remaining two studies did not conduct kinematic analyses [52, 62]. Only two studies [58, 61] explicitly stated which kinematic model was used. Joint kinematics were the most common focus, particularly ankle and foot mechanics in the sagittal plane (n = 11, foot strike angle/index [53, 55, 57, 58, 60, 62], discrete sagittal angles [55, 56, 58–60, 64], time series [61] or stability of the angles based on maximum Lyapunov exponent (MLE) [63]. Knee and hip mechanics were analyzed in eight [53, 55, 56, 58–61, 63] and five [53, 58, 59, 61, 63] studies, respectively. Frontal plane movements were investigated in six studies [54–56, 59, 61, 64], with ankle frontal angle analyzed in all. Other parameters included tibialis movements [55, 56, 58], head acceleration [55, 56], dynamic stability of body segments based on MLE [61], COM movement [52–54, 61], and leg/global stiffness [52, 58, 61, 62] or joint (ankle and knee) stiffness [58, 60]. GRF was analyzed in six studies [52, 54, 57, 58, 60, 64], and joint moment/work in three [51, 53, 58]. Running economy was assessed in one study [59].

Given the limited availability of effect sizes and the substantial heterogeneity in outcome variables and experimental designs, a semi-quantitative synthesis was conducted. Table 7 summarizes effect direction, statistical significance, and sole thickness contrasts across all included studies.

### Statistical Analysis

The majority of studies [53–61, 64] used repeated measures ANOVA/Friedman tests for shoe comparisons, with *post-hoc* tests in most cases either with [53, 54, 57, 59–61, 64] or without [55, 56, 58] corrections for multiple tests. Other methods included a mixed general linear model [63]; Spearman correlations [62]; and principal component analysis and clustering [52]. Only five studies reported effect sizes [55, 57, 60, 61, 64].

### Outcomes

In the following sections, shoes are abbreviated as “S” followed by their heel thickness in mm (e.g., S3 for shoes with a heel thickness of 3 mm). If heel thickness was not reported, midsole thickness is used instead and marked with an asterisk (e.g., S25* for shoes with a midsole thickness of 25 mm).

#### Spatiotemporal

Stance time was longer for thicker soles in five studies, although not all pairwise shoe comparisons were significant [53, 55–58]. Chambon et al. [58] found that S16 resulted in a longer stance time than S0, while Law et al. [57] observed longer stance times for S9*, S21*, S25*, and S29* compared to S1*. TenBroek et al. found that S14 and S24 led to longer stance time than S3 in a 6-min run [56], while S24 resulted in a longer stance time than both S3 and S14 in a 30-min run [55]. Conversely, Barrons et al. [59] found no significant difference between shoes. Horvais and Samozino [62] found no correlations between heel thickness and stance time. Koegel et al. [52] conducted a clustering analysis to classify runners based on their responses to different shoes and found that participants were grouped into three distinct clusters, each exhibiting unique response patterns to increasing sole thickness.

Stride frequency, normalized to leg length, was higher in S50 compared to S35 at 4.2 m/s but not at 2.8 m/s [61]. Barrons et al. [59] and Law et al. [57] found no significant differences in stride frequency, and Horvais and Samozino [62] reported no significant correlations with heel thickness. Koegel et al. [52] found that step frequency had the smallest contribution to forming runner clusters and, therefore, did not analyze its variation across shoes and clusters in detail.

Stride length showed no significant differences between shoes [57, 59]. Koegel et al. [52] did not analyze stride length in detail due to its small contribution to cluster formation.

Duty factor showed no difference between shoes in Kettner et al. [61], and there was no significant correlation with heel thickness in Horvais and Samozino [62]. Flight time also showed no significant correlations with heel thickness [62].

Other parameters, including detrended fluctuation of stride time [61], ratio of braking to propulsion duration [61], or time to complete the turn task [54] did not show any significant differences between shoes. Koegel et al. [52] found no significant differences in running speed across clusters.

#### Joint Kinematics in the Sagittal Plane

Three studies [55, 56, 64] reported greater ankle dorsiflexion at IC with thicker soles, though not all comparisons were significant. Hannigan and Pollard [64] found more dorsiflexion with S22 than S10. TenBroek et al. [56] observed greater dorsiflexion with S14 and S24 compared to S3, and their second study [55] found greater dorsiflexion with S24 than S3 and S14. Barrons et al. [58, 59] found no significant differences. Law et al. [57] reported a higher foot strike angle, indicating a stronger rearfoot strike pattern with S25* compared to S1*, while Horvais and Samozino [62] reported a positive correlation between sole thickness and foot strike angle. Zhang et al. [60] found lower foot strike angles with S42* and S54* than S30*. Chambon et al. [58] found no differences in foot strike angle. Two studies found no differences at toe off (TO) [59, 64].

For peak dorsiflexion, Barrons et al. [59] reported lower values with S50 than S35 and S40, while Miyazaki et al. [53] reported higher values with S35 than S24. Hannigan and Pollard [64] found no differences. Zhang et al. [60] found the lowest dorsiflexion velocity with S54*. Three studies [58, 60, 64] examined ankle range of motion (ROM) during stance, but only Hannigan and Pollard [64] reported significant differences. Kettner et al. [61] found no significant differences in the sagittal ankle angle time series. Frank et al. [63] reported no significant shoe effects on dynamic stability of the sagittal ankle angle.

The sagittal knee angle at IC showed mixed results [55, 56, 58, 59]. TenBroek et al. [56] found more knee flexion with S14 than S24, while their other study [55] reported more knee flexion with S14 and S24 than S3. Barrons et al. [59] and Chambon et al. [58] detected no significant effects. Two studies [57, 59] found no differences in peak knee flexion, and Zhang et al. [60] reported no effects on knee flexion velocity. Two studies found differences in knee ROM [55, 56], while two others found no effects [58, 60]. Kettner et al. [61] reported no differences in the sagittal knee angle time series. Frank et al. [63] found no differences in dynamic stability of the sagittal knee angle.

No differences in sagittal hip angle at IC [58, 59] or TO [59] were reported. Hip ROM during stance [58] and the entire hip angle time series [61] showed no significant differences. Frank et al. [63] found no differences in dynamic stability of the sagittal hip angle.

The sagittal thigh angle at IC showed mixed results. S14 and S24 led to greater thigh flexion than S3 in one study [55], while only S14 resulted in more flexion in another [56].

Only one study quantified dynamic landing variables (i.e., shank retraction velocity, heel velocity, and ankle angular velocity) at or immediately prior to IC [60], whereas the remaining studies focused on discrete joint angles and did not report dynamic initial landing conditions.

#### Joint Kinematics in the Frontal Plane

Four studies [55, 56, 61, 64] reported no significant differences in ankle angles at IC across shoe conditions. Three studies [59, 61, 64] found a trend toward greater eversion with thicker soles, though not all pairwise comparisons were significant. Barrons et al. [59] found greater peak eversion with S45 than S35. Hannigan andPollard [64] reported higher peak eversion with S33 than S22. Kettner et al. [61] detected higher peak eversion with S50 than S35. TenBroek et al. [55] found lower peak eversion with S24 compared to S3 and S14. Barrons et al. [54] reported no significant differences in peak inversion or frontal angles during running turns.

Foot ROM in the frontal plane showed no significant differences in two studies [56, 64], while TenBroek et al. [55] found greater ROM with S14 than S24. Eversion duration was greater with thicker soles in Kettner et al. [61] (S50 > S35) and in Hannigan and Pollard [64] (S33 > S10 and S33 > S22). Kettner et al. [61] also reported greater foot inversion with S35 than S50 and found no differences in knee and hip frontal angle time series.

#### Segment Kinematics

TenBroek et al. [55, 56] found reduced tibial internal rotation ROM with thicker soles (S14 < S3, S24 < S3). Chambon et al. [58] found no shoe effects on tibial peak acceleration, while TenBroek et al. [55, 56] reported lower tibial peak accelerations with thicker soles. Thicker soles also resulted in lower peak head accelerations (S24 < S3 in two studies [55, 56]: S24 < S14 in one study, S14 < S3 in the other [55]). The transfer function, which evaluated shock attenuation using head and tibial accelerations, showed no shoe effects. Finally, Kettner et al. [61] found no significant differences in the dynamic local stability of head, trunk, hip, or foot segments.

#### Center of Mass Movement

Kettner et al. [61] found higher vertical COM oscillation with thicker soles (S50 > S35), while Miyazaki et al. [53] detected no significant differences. Koegel et al. [52] observed that vertical COM oscillation was the second most influential factor in forming clusters, with different response patterns to increasing sole thickness. Barrons et al. [54] found no significant shoe effects on peak COM velocities during running turns.

#### Stiffness

Zhang et al. [60] reported the highest ankle and knee stiffness with the thickest sole (S54* > S40* and S54* > S30*), while Chambon et al. [58] found no significant effects. Leg stiffness was unaffected by shoe conditions in Kettner et al. [61], whereas Horvais and Samozino [62] found lower leg stiffness with greater heel thickness. Koegel et al. [52] reported that vertical stiffness was the most influential factor in their clustering analysis, with varied responses to increasing sole thickness.

#### Joint Kinetics and Energetics

Reinschmidt and Nigg [51] found that each 10 mm increase in heel thickness increased the maximum plantarflexion moment by 6.3 Nm. In contrast, Chambon et al. [58] found no significant effects on maximum plantarflexion, knee flexion, or hip flexion moments. Miyazaki et al. [53] found that S35 led to higher peak plantar torque, positive ankle work and negative knee work compared to S24.

#### Ground Reaction Forces

Three out of four studies found lower vertical GRF loading rates with thicker soles, though not all comparisons were significant. Hannigan and Pollard [64] and Law et al. [57] reported lower loading rates for thicker soles (Table 7). Zhang et al. [60] found higher loading rates with the thickest sole (S54* > S40* and S54* > S30*). Peak vertical GRF showed no significant differences in three studies [54, 58, 64], while Barrons et al. [54] found lower peak propulsive GRF with S50 during running turns.

#### Running Economy and Effective Leg Length

Barrons et al. [59] reported no effects on average VO_2_ or energetic cost. However, they reported increased effective leg length for thicker soles in most comparisons, with significant increases in leg length at IC, mid-stance, and TO for thicker soles (e.g., S50 > S35, S50 > S40).

## Discussion

To date, no review has focused on studies in which sole thickness was the primary variable of interest. This systematic review aimed to address this gap by examining how sole thickness impacts spatiotemporal variables, kinematics, kinetics, and running economy. This review of 14 studies revealed a consistent trend of longer stance times with thicker soles, although other spatiotemporal parameters showed no clear pattern. Thicker soles generally resulted in greater dorsiflexion at IC in the sagittal plane, with fewer effects on knee and hip kinematics. In the frontal plane, there was a weaker trend toward greater peak eversion with thicker soles. Joint kinetics, stiffness parameters, and COM movement showed no clear trends. While vertical GRF peaks remained largely unchanged, the loading rate tended to decrease with increasing sole thickness. Running economy, examined in only one study, showed no significant effects of shoe thickness.

The interpretation of these findings is limited by recurrent methodological constraints, including small sample sizes, lack of a priori power calculations, heterogeneous experimental protocols, incomplete reporting of shoe characteristics, and the acute laboratory-based nature of the included studies. These factors contribute to substantial uncertainty and likely explain the inconsistent findings observed across several outcome domains. Consequently, the certainty of evidence for most biomechanical responses to changes in sole thickness should be considered low.

### Outcomes

#### Spatiotemporal

Stance time tended to increase with thicker soles [53, 55–58], typically when the thickness difference was ≥ 8 mm, though not all increases led to significant changes [53, 55–59]. The increase in stance time with thicker soles may be attributed to the time required for midsole material deformation—the greater the amount of material, the longer the deformation process, leading to an extended stance phase [58]. However, this increase in stance time did not correspond to modulated leg stiffness [58, 62], as one might expect [58, 66]. One study found no significant difference in stance time despite a 15 mm thickness difference [59], likely due to the greater compliance (i.e., deformation under load) in AFT shoes [7].

Interestingly, longer stance times with thicker soles did not translate into changes in step frequency or stride length [57]. However, it should be noted that only two studies compared stance time alongside other spatiotemporal parameters across different shoes [57, 59].

#### Joint and Segment Kinematics

The most notable shoe effects were observed in sagittal ankle kinematics, particularly an increase in dorsiflexion at IC with thicker soles [55, 56, 64]. In contrast, the effects on knee and hip kinematics were inconsistent and less pronounced across studies [55, 56, 58, 59]. This discrepancy may be explained by the fact that shoe modifications directly influence the ankle joint, leading to compensatory adjustments being made primarily at the ankle rather than at the knee or hip. Despite the lack of significant knee and hip joint angle changes, thicker soles still led to increased vertical oscillation of the COM [61], suggesting that the motor control system responded to differences in sole thickness, as COM regulation is a key aspect of running mechanics [67].

Findings on sagittal foot strike angle varied between studies. Law et al. [57] and Horvais and Samozino [62] observed a stronger rearfoot strike pattern (i.e., a higher foot strike angle) with increasing sole thickness, while Zhang et al. [60] found that participants tended to adopt a more midfoot strike pattern. These discrepancies may stem from the different ranges of sole thickness examined. In Law et al. [57] and Horvais and Samozino [62], sole thickness varied between 1–25 mm and 0–25 mm, respectively, whereas Zhang et al. [60] tested shoes with thicknesses ranging from 30 to 54 mm. This suggests that the relationship between foot strike angle and sole thickness may follow a U-shaped pattern, with peak modulation occurring around 30 mm.

In the frontal plane, ankle kinematics at IC showed no significant differences between shoes. However, a tendency for greater peak eversion with thicker soles was observed [59, 61, 64], which has been interpreted as a sign of reduced ankle stability. That said, not all pairwise shoe comparisons were significant. For example, Barrons et al. [59] reported that S45 resulted in greater eversion than S35, but S50 did not differ from S35. Similarly, Hannigan and Pollard [64] found that S33 led to greater peak eversion than S22 but did not differ from S10. Therefore, generalizing these results as evidence of reduced ankle stability or increased injury risk with thicker soles would be premature.

Lastly, analyzing non-sagittal kinematics is challenging due to modeling difficulties and measurement errors [68]. Differences across platforms (e.g., OpenSim vs. Anybody) [69] and calculation methods [70] make it difficult to determine the most accurate model, so results should be interpreted carefully.

Importantly, landing mechanics are defined not only by joint posture at IC but also by dynamic variables such as segment and joint velocities [60]. Because most studies reported only joint angles at IC (Table 7), dynamic landing conditions were implicitly assumed to be comparable across shoe conditions. However, changes in sole thickness may alter the velocity- and acceleration-related characteristics of the foot or shank at ground contact, even when joint angles at IC appear similar.

#### Center of Mass

The effects of sole thickness on vertical oscillation of the COM were inconsistent results. Kettner et al. [61] found that a thicker sole (S50) led to greater vertical oscillation compared to a thinner sole (S35). However, Miyazaki et al. [53] reported no significant differences in vertical oscillation between different thicknesses. Koegel et al. [52] highlighted that vertical oscillation of the COM was influential in clustering runners based on their biomechanical responses to sole thickness, but the three identified clusters displayed distinct patterns of adaptation. These discrepancies may be attributed to differences in measurement techniques. Kettner et al. [61] used a full-body kinematic model with gold-standard motion capture, while Miyazaki et al. [53] used a lower-body model, which could explain the differing results. Use of a commercial wearable sensor, which has not been validated for measuring vertical COM oscillation, in Koegel et al. [52] introduces additional uncertainty. Given the importance of COM movement in running mechanics [67], further research with standardized methods is needed to confirm whether thicker soles consistently lead to increased vertical oscillation of the COM.

#### Stiffness

The relationship between sole thickness and various biomechanical stiffness values (including ankle, knee, leg, and vertical stiffness) was inconsistent across studies, suggesting a complex interaction. These contradictions can be partially explained by differences in stiffness estimation methods. Zhang et al. [60] assessed ankle and knee stiffness using purely kinematic data and found that ankle stiffness increased with thicker soles (S54* > S42* > S30*), with knee stiffness also highest in the thickest sole. Conversely, Chambon et al. [58] incorporated both force and kinematic data and found no significant effect of sole thickness on ankle or knee stiffness. Using both kinetic and kinematic data, these authors reported no significant effects on vertical stiffness. However, Koegel et al. [52] estimated vertical stiffness using only kinematic data and found that it was a key factor in clustering runners into distinct response groups. Their study suggested that individual runners exhibit different adaptations to increasing sole thickness. These findings highlight the need for a standardized approach to measuring stiffness, as variations in methodology can lead to conflicting results.

#### Joint Kinetics and Energetics

A few studies [51, 53, 58] investigated joint moments, torques, or work, and their findings varied. Reinschmidt and Nigg [51] reported an increased maximum plantarflexion moment with a temporal shift in the stance phase, though their small sample size and lack of foot strike pattern data limited the reliability of their results. Additionally, the different heel-to-toe drops of the shoes tested could have confounded the sole thickness effects [45]. On the other hand, Chambon et al. [58] tested shoes with a standardized heel-to-toe drop (0 mm) and found no significant effects of sole thickness on plantarflexion, knee flexion, or hip flexion moments. This suggests that the changes observed by Reinschmidt and Nigg [51] might have been influenced by the heel-to-toe drop rather than sole thickness. Miyazaki et al. [53] found that a thicker sole (S35 vs. S24) increased peak plantar torque, positive ankle work, and negative knee work, while decreasing peak knee extension torque. However, this study also involved varying heel-to-toe drops, complicating the attribution of effects solely to sole thickness. In summary, the effects of sole thickness on joint kinetics and energetics remain unclear due to the methodological differences and limited number of studies.

#### Ground Reaction Forces

The loading rate of vertical GRF generally decreased with increased sole thickness [52, 54, 64], with one study finding no significant shoe effects [58]. This finding can be explained by the increased cushioning and reduced stiffness of the shoes [52, 59]. GRF parameters are often analyzed to understand shock attenuation and potential injury risk. A systematic review suggested that runners with a history of stress fractures tend to have greater loading rates than those without prior running injuries [71]. However, a more recent study [72] argued that vertical loading rate is not directly associated with running injuries, making the protective role of thicker soles uncertain.

#### Running Economy and Leg Length

Running economy was investigated in only one study [59], which is surprising given the ongoing debate on the effects of sole thickness on performance. Some studies suggest that greater sole thickness increases effective leg length, which may enhance stride length and running economy [4]. However, not all studies support this hypothesis [6]. The findings of Barrons et al. [59] also contradicted this, as they observed an increase in estimated leg length with thicker soles, but this did not translate into longer stride length or improved running economy.

### Individual Responses to the Different Shoes

While several studies reported non-significant mean effects of sole thickness on key outcomes (e.g., step frequency), evidence indicates that individual responses can vary substantially. Koegel et al. [52] demonstrated that runners can be classified into distinct response clusters when midsole thickness is modified, with differences between clusters primarily related to changes in vertical stiffness, and vertical oscillation of COM. Evidence from broader footwear literature further supports that the biomechanical responses to footwear modifications are highly individual and context-dependent [10] Furthermore, pronounced inter-individual variability in responses to advanced footwear technologies has been reported in both elite and recreational runners, suggesting that habitual running characteristics may influence how individuals respond, or do not respond, to different running shoes [47]. Together, these findings suggest that inter-individual variability may obscure systematic effects at the group level and should be considered when interpreting biomechanical responses to altered sole thickness.

### Range of Sole Thickness Across Studies

Although no universally accepted classification of shoe sole thickness exists, several influential lines of research implicitly distinguish between different thickness regimes. Studies comparing minimal and conventional footwear primarily focus on sensory input, collision mechanics, and foot strike adaptations [12, 17], whereas others have explicitly contrasted conventional shoes with highly cushioned designs, emphasizing nonlinear metabolic and mechanical trade-offs associated with increased midsole thickness and mass [27, 73]. In addition, the 40 mm stack-height limit imposed by World Athletics represents a clear regulatory and design threshold that has further reinforced the distinction between moderately thick and exaggerated sole constructions [4, 8]. Within this context, sole thickness ranges can be considered pragmatically as minimal-to-traditional (0–25 mm), traditional-to-moderately thick (25–40 mm), and moderately thick-to-exaggerated (> 40 mm) to facilitate range-specific interpretation of existing evidence rather than to imply strict sole thickness thresholds.

Biomechanical responses to shoe sole thickness changes are range-specific rather than continuous across the full thickness spectrum. In studies examining comparisons that included minimal and traditional footwear conditions (up to 25 mm), several authors reported changes in foot strike characteristics, such as higher foot strike angles or a stronger rearfoot strike tendency with increasing sole thickness [57, 62]. However, these effects were not universal, as other studies spanning similar thickness ranges reported no significant changes in foot strike angle or strike index [58]. Across studies comparing shoes with greater versus lesser sole thickness, stance time often increased in thicker conditions, although these effects were typically observed in experimental designs spanning broad thickness differences (≥ 8 mm) rather than being confined to a specific transition or sole thickness category [53, 55–58]. Within these comparisons, several studies also reported greater ankle dorsiflexion at IC or during stance in thicker conditions [55, 56, 64], which may reflect altered midsole deformation and cushioning behavior during early stance. Evidence related to exaggerated sole thicknesses (> 40 mm) remains limited and is currently based on a small number of studies. In this range, Barrons et al. [54] and Kettner et al. [61] reported changes in frontal-plane ankle mechanics, particularly greater peak eversion with thicker shoes, whereas other ankle kinematics outcomes showed no consistent differences. Similarly, vertical COM oscillation was increased in one study using exaggerated sole thicknesses [61], but no effect was observed in COM velocity [54]. Collectively, these findings indicate that biomechanical responses at higher sole thicknesses are more variable and may reflect interactions between increased cushioning, shoe mass, and altered stability demands, although direct evidence for these mechanisms remains limited.

### Measurement and Reporting Protocol of Shoes

World Athletics uses the term "sole thickness" [8] which is measured at both the forefoot and heel. In contrast, scientific studies often use the terms “midsole thickness” [53, 74, 75] or “stack height” [5, 52, 76, 77], although they typically refer to the same feature. To maintain consistency with World Athletics regulations, this review uses the term "sole thickness".

According to the latest World Athletics regulations [8], sole thickness should be measured at the center of the forefoot and the center of the heel, which are specifically defined as 12% and 75% of the internal shoe length, respectively. However, only one study explicitly reported where these measurements were taken [61]. Additionally, some studies reported only midsole thickness [52, 57, 60] or heel thickness [51, 59], making it more difficult to interpret results due to missing information (e.g., heel-to-toe drop).

Shoe mass represents another potential confounding factor, depending on the difference in mass between tested shoes. A mass increase of 100 g has been shown to negatively impact running economy and performance, whereas a 50 g difference had no effect [78]. In studies where mass was explicitly reported, the difference between tested shoes was generally below 50 g. However, shoe mass was not reported in all studies [51, 57, 58, 62–64], further complicating result interpretation.

The material composition of shoe foam is another important factor influencing running economy and biomechanics [79]. However, not all reviewed studies explicitly stated which materials were used in the tested shoes [51, 60–64].

Importantly, changes in sole thickness were often accompanied by variations in other shoe characteristics, particularly in AFT shoes, where sole thickness is inherently linked to midsole material properties and plate geometry. Therefore, the reported effects should be interpreted as the influence of sole thickness within specific shoe designs rather than as fully isolated effects. For better transparency, future studies should report the full midsole geometry (including forefoot and heel thickness with precise measurement locations and heel-to-toe drop), shoe mass, and material composition.

### Limitations and Future Directions

#### Shoes

The shoes used in the reviewed studies mainly differed in sole thickness, with some studies also reporting slight variations in shoe mass [52–56, 60, 61]. Two studies accounted for mass differences by adding small weights to the shoes [59, 60], while others did not match mass between shoes, making it challenging to fully isolate the effects of sole thickness. Additionally, in some studies, the heel-to-toe drop varied between shoes [53, 55, 56, 60, 63], which could have been another confounding factor influencing the results.

Four studies involved AFT shoes. Given that these regulations came after the AFT era, further research on AFT shoes is needed to better understand how sole thickness interacts with other shoe features [7].

In addition, the reviewed studies investigated different ranges and magnitudes of sole thickness, and these were not consistent across the literature. Some studies focused on minimal-to-traditional footwear (0–25 mm), others compared traditional to moderately thick soles (25–40 mm), and only a small number examined exaggerated sole thicknesses (> 40 mm).

#### Participants

The studies reviewed primarily included recreational to experienced runners, with a notable underrepresentation of elite and novice runners. Additionally, most participants were habitual rearfoot strikers, and some studies did not control for this factor. To provide a more comprehensive understanding of the impact of sole thickness, future research should involve elite and novice runners, as well as other types of strikers.

Most participants were male, with a mean height range of 1.69 to 1.81 m and mean mass between 58.3 and 73.6 kg. To ensure a more representative sample, future studies should include a broader range of participants, particularly females, and those outside typical height and mass ranges.

#### Study Design

Only four studies performed a priori power calculations to determine sample size, which may have limited the statistical power of their findings and raises the possibility of small-study effects and publication bias. The limited reporting of effect sizes and quantitative outcome data across studies represents a major limitation of the current evidence base and restricts the feasibility of standardized quantitative synthesis. The majority of studies (n = 13) were conducted in laboratory settings, using either a standard laboratory floor or a motorized treadmill for running tests (Table 5). However, since ground surface material and stiffness can affect running economy and biomechanics [80–82], future research should incorporate various surfaces (e.g., track or concrete) to better simulate real-life running conditions.

One further limitation relates to running speed, which varied across studies and was not always consistently reported or systematically manipulated (Table 5). Most studies examined a single, submaximal running speed, and only a limited number explored more than one speed within the same experimental design. Because running speed influences stance dynamics, and joint kinematics, the effects of sole thickness may differ across running speeds, limiting the generalizability of the reported findings [83, 84].

A further limitation concerns the limited reporting of dynamic initial conditions of landing. While most studies quantified joint posture at IC, variables describing landing dynamics, such as segment velocities or joint angular velocities, were rarely reported (Table 7). Because these dynamic variables contribute to impact mechanics and early stance behavior [60], their omission restricts the interpretation of how sole thickness influences landing mechanics across studies.

Another limitation was the inconsistency in biomechanical modeling, with an overreliance on lower-body models: only two studies used full-body models (Table 5). Additionally, only two studies explicitly stated their kinematical model, making cross-study comparisons challenging. Another limitation was that most studies focused on a single joint degree of freedom, overlooking coordination of multiple degrees of freedom which is crucial for understanding motor control [85].

Recent literature suggests that individualized shoe design and development could be beneficial in the future [10]. In line with this, future research could explore more personalized approaches, such as clustering participants based on their response to specific shoe features [52]. Several studies included in the present review reported heterogeneous or inconsistent biomechanical responses to changes in sole thickness, indicating that runners may adopt different individual response strategies when interacting with shoe cushioning. In this context, additional measures such as running economy [86], muscle activity [87, 88], and movement coordination [89] remain underexplored but may help to explain how runners redistribute loads and adapt neuromuscular control across different sole thickness conditions. To provide a more comprehensive understanding of the effects of sole thickness, further studies should address these aspects.

## Conclusion

A review of 14 studies on the effects of running shoe sole thickness within shoe designs revealed consistent trends, including longer stance times, increased ankle dorsiflexion at IC, and a decreased loading rate of GRF with thicker soles. However, many other parameters, such as step frequency, knee kinematics, and stiffness, did not show consistent trends across studies. Shoe mass and heel-to-toe drop emerged as potential confounding factors that may have influenced the results, and the available evidence is further limited by small sample sizes, predominantly male cohorts, and acute laboratory-based study designs. Overall, the findings of this review indicate that biomechanical responses to changes in shoe sole thickness are dependent on the magnitude of the thickness change and the experimental conditions and should be interpreted with caution given the low to very low certainty of evidence. The reported effects reflect acute biomechanical responses observed under controlled laboratory conditions and are not uniform across studies or the full thickness spectrum. This suggests that increasing sole thickness does not lead to predictable biomechanical adaptations across all runners or footwear designs. A likely explanation is that changes in sole thickness alter foot–ground interaction and midsole deformation, thereby influencing stance dynamics and joint-level control in different ways depending on the individual runner characteristics and the overall shoe design. Given that all included studies assessed acute biomechanical responses during short-term laboratory experiments, no conclusions can be drawn regarding injury risk, long-term adaptation, or sustained performance effects of increased sole thickness. Future research should aim to report shoe features more comprehensively and transparently, include a more diverse range of participants (e.g., female runners, forefoot strikers), and broaden the analysis to include aspects like running economy, muscle activity, and movement coordination. This would provide a more thorough understanding of the effects of sole thickness, within complex shoe designs, on running economy and biomechanics.

## Data Availability

All data generated or analyzed during this study are included in this published article.

## Abbreviations

AFT: Advanced footwear technologies
COM: Center of mass
IC: Initial contact
GRF: Ground reaction force
MLE: Maximum Lyapunov exponent
PERSiST: Prisma in exercise, rehabilitation, sport medicine and sports science
PICO: Population, intervention, comparison, outcome, and study design
PRISMA: Preferred reporting items for systematic reviews and meta-analyses
RoB-2: Cochrane risk of bias instrument tool
ROM: Range of motion
TO: Toe-off

## References

[1] Honert EC, Mohr M, Lam WK, Nigg S. Shoe feature recommendations for different running levels: a Delphi study. PLoS ONE. 2020;15(7):e0236047. 10.1371/journal.pone.0236047. 32673375 10.1371/journal.pone.0236047 PMC7365446

[2] Cheung RTH, Wong RYL, Chung TKW, Choi RT, Leung WWY, Shek DHY. Relationship between foot strike pattern, running speed, and footwear condition in recreational distance runners. Sports Biomech. 2017;16:238–47. 10.1080/14763141.2016.1226381. 27593384 10.1080/14763141.2016.1226381

[3] Zhou W, Yin L, Jiang J, Zhang Y, Hsiao CP, Chen Y, et al. Surface effects on kinematics, kinetics and stiffness of habitual rearfoot strikers during running. PLoS ONE. 2023;18(3):e0283323. 10.1371/journal.pone.0283323. 36947495 10.1371/journal.pone.0283323 PMC10032480

[4] Burns GT, Tam N. Is it the shoes? A simple proposal for regulating footwear in road running. Br J Sports Med. 2020;54:439–41. 10.1136/bjsports-2018-100480. 31630088 10.1136/bjsports-2018-100480

[5] Ruiz-Alias SA, Jaén-Carrillo D, Roche-Seruendo LE, Pérez-Castilla A, Soto-Hermoso VM, García-Pinillos F. A review of the potential effects of the World Athletics stack height regulation on the footwear function and running performance. Appl Sci. 2023;13:11721. 10.3390/app132111721.

[6] Hoogkamer W. More isn’t always better. Footwear Sci. 2020;12:75–7. 10.1080/19424280.2019.1710579.

[7] Burns GT, Joubert DP. Running shoes of the postmodern footwear era: a narrative overview of advanced footwear technology. Int J Sports Physiol Perform. 2024. 10.1123/ijspp.2023-0446. 39117307 10.1123/ijspp.2023-0446

[8] World Athletics Council. Book C - C2.1A athletic shoe regulations, book of rules. World Athletics; 2022. https://worldathletics.org/about-iaaf/documents/book-of-rules. Accessed 31 Jan 2025

[9] Davis IS. The re-emergence of the minimal running shoe. J Orthop Sports Phys Ther. 2014;44:775–84. 10.2519/jospt.2014.5521. 25211531 10.2519/jospt.2014.5521

[10] Mai P, Robertz L, Robbin J, Bill K, Weir G, Kurz M, et al. Towards functionally individualised designed footwear recommendation for overuse injury prevention: a scoping review. BMC Sports Sci Med Rehabil. 2023;15:1–20. 10.1186/s13102-023-00760-x. 37951935 10.1186/s13102-023-00760-x PMC10638717

[11] Nigg BM, Baltich J, Hoerzer S, Enders H. Running shoes and running injuries: mythbusting and a proposal for two new paradigms: “Preferred movement path” and “comfort filter.” Br J Sports Med. 2015;49:1290–4. 10.1136/bjsports-2015-095054. 26221015 10.1136/bjsports-2015-095054

[12] Lieberman DE, Venkadesan M, Werbel WA, Daoud AI, Dandrea S, Davis IS, et al. Foot strike patterns and collision forces in habitually barefoot versus shod runners. Nature. 2010;463:531–5. 10.1038/nature08723. 20111000 10.1038/nature08723

[13] Hollander K, Argubi-Wollesen A, Reer R, Zech A. Comparison of minimalist footwear strategies for simulating barefoot running: a randomized crossover study. PLoS ONE. 2015;10(5):e0125880. 10.1371/journal.pone.0125880. 26011042 10.1371/journal.pone.0125880 PMC4444250

[14] Izquierdo-Renau M, Sanchis-Sanchis R, Priego-Quesada JI, Encarnación-Martínez A, Queralt A, Pérez-Soriano P. Effects of minimalist footwear and foot strike pattern on plantar pressure during a prolonged running. Appl Sci. 2022;12:506. 10.3390/app12010506.

[15] Rixe JA, Gallo RA, Silvis ML. The barefoot debate: can minimalist shoes reduce running-related injuries? Curr Sports Med Rep. 2012. 10.1249/jsr.0b013e31825640a6. 22580495 10.1249/JSR.0b013e31825640a6

[16] Perkins KP, Hanney WJ, Rothschild CE. The risks and benefits of running barefoot or in minimalist shoes: a systematic review. Sports Health. 2014;6:475–80. 10.1177/1941738114546846. 25364479 10.1177/1941738114546846 PMC4212355

[17] Hollander K, Davis IS. Minimal shoes: restoring natural running mechanics. In: Ledoux WR, Telfer S, editors. Foot and Ankle Biomechanics. Academic Press, Cambridge . 2023.

[18] Giuliani J, Masini B, Alitz C, Owens BD. Barefoot-simulating footwear associated with metatarsal stress injury in 2 runners. Orthopedics. 2011;34:320–3. 10.3928/01477447-20110526-25. 10.3928/01477447-20110526-25 21717998

[19] Ridge ST, Johnson AW, Mitchell UH, Hunter I, Robinson E, Rich BSE, et al. Foot bone marrow edema after a 10-wk transition to minimalist running shoes. Med Sci Sports Exerc. 2013;45:1363–8. 10.1249/MSS.0b013e3182874769. 23439417 10.1249/MSS.0b013e3182874769

[20] Bermon S, Garrandes F, Szabo A, Berkovics I, Adami PE. Effect of advanced shoe technology on the evolution of road race times in male and female elite runners. Front Sports Act Living. 2021;3:653173. 10.3389/fspor.2021.653173. 33969296 10.3389/fspor.2021.653173 PMC8100054

[21] Rodrigo-Carranza V, González-Mohíno F, Santos del Cerro J, Santos-Concejero J, González-Ravé JM. Influence of advanced shoe technology on the top 100 annual performances in men’s marathon from 2015 to 2019. Sci Rep. 2021;11:22458. 10.1038/s41598-021-01807-0. 34789828 10.1038/s41598-021-01807-0 PMC8599511

[22] Hoogkamer W, Kram R, Arellano CJ. How biomechanical improvements in running economy could break the 2-hour marathon barrier. Sports Med. 2017;47(9):1739–50. 10.1007/s40279-017-0708-0. 28255937 10.1007/s40279-017-0708-0

[23] Weiss M, Newman A, Whitmore C, Weiss S. One hundred and fifty years of sprint and distance running – past trends and future prospects. Eur J Sport Sci. 2016;16:393–401. 10.1080/17461391.2015.1042526. 26088705 10.1080/17461391.2015.1042526 PMC4867877

[24] Caesar E. Two hours: the quest to run the impossible marathon. London: Penguin UK; 2020.

[25] Rodrigo-Carranza V, González-Mohíno F, Santos-Concejero J, González-Ravé JM. The effects of footwear midsole longitudinal bending stiffness on running economy and ground contact biomechanics: a systematic review and meta-analysis. Eur J Sport Sci. 2022;22:1508–21. 10.1080/17461391.2021.1955014. 34369282 10.1080/17461391.2021.1955014

[26] Ortega JA, Healey LA, Swinnen W, Hoogkamer W. Energetics and biomechanics of running footwear with increased longitudinal bending stiffness: a narrative review. Sports Med. 2021;51:873–94. 10.1007/s40279-020-01406-5. 33830444 10.1007/s40279-020-01406-5

[27] Hoogkamer W, Kipp S, Frank JH, Farina EM, Luo G, Kram R. A comparison of the energetic cost of running in marathon racing shoes. Sports Med. 2018;48:1009–19. 10.1007/s40279-017-0811-2. 29143929 10.1007/s40279-017-0811-2 PMC5856879

[28] World Athletics Council. International Association of Athletics Federations Competition Rules 2018–2019. 2017. https://worldathletics.org/about-iaaf/documents/book-of-rules. Accessed 26 Jan 2025

[29] World Athletics Council. Amendments to Rule 5 of the Technical Rules 2020. https://worldathletics.org/search?q=Amendmentto%20Rule%205%20of%20the%20Technical%20Rules. Accessed 7 Mar 2025

[30] Hannigan JJ, Westley L, Jin L. Injury and performance-related running biomechanics in advanced footwear technology compared to minimalist footwear. Footwear Sci. 2024;16:115–21. 10.1080/19424280.2024.2327317.

[31] Bonato M, Marmondi F, Faelli EL, Pedrinelli C, Ferraris L, Filipas L. Advanced footwear technology in non-elite runners: a survey of training practices and reported outcomes. Sports Basel. 2024;12:356. 10.3390/sports12120356. 39728896 10.3390/sports12120356 PMC11679133

[32] Rodrigo-Carranza V, Hoogkamer W, González-Ravé JM, González-Mohíno F. Relationship between advanced footwear technology longitudinal bending stiffness and energy cost of running. Scand J Med Sci Sports. 2024. 10.1111/sms.14687. 38923087 10.1111/sms.14687

[33] Riedl M, von Diecken C, Ueberschär O. One shoe to fit them all? Effect of various carbon plate running shoes on running economy in male and female amateur triathletes and runners at individual training and race paces. Appl Sci Basel. 2024;14:11535. 10.3390/app142411535.

[34] Heyde C, Nielsen A, Roecker K, Godsk Larsen R, de Zee M, Kersting U, et al. The percentage of recreational runners that might benefit from new running shoes. A likely scenario. Footwear Sci. 2022;14:163–72. 10.1080/19424280.2022.2095042.

[35] Patoz A, Lussiana T, Breine B, Gindre C. The Nike Vaporfly 4 %: a game changer to improve performance without biomechanical explanation yet. Footwear Sci. 2022;14(3):147–50. 10.1080/19424280.2022.2077844.

[36] Hébert-Losier K, Pamment M. Advancements in running shoe technology and their effects on running economy and performance–a current concepts overview. Sports Biomech. 2023;22:335–50. 10.1080/14763141.2022.2110512. 35993160 10.1080/14763141.2022.2110512

[37] Joubert DP, Oehlert GM, Jones EJ, Burns GT. Comparative effects of advanced footwear technology in track spikes and road-racing shoes on running economy. Int J Sports Physiol Perform. 2024;19:705–11. 10.1123/ijspp.2023-0372. 38815961 10.1123/ijspp.2023-0372

[38] Hoitz F, Mohr M, Asmussen M, Lam WK, Nigg S, Nigg B. The effects of systematically altered footwear features on biomechanics, injury, performance, and preference in runners of different skill level: a systematic review. Footwear Sci. 2020;12:193–215. 10.1080/19424280.2020.1773936.

[39] Page MJ, McKenzie JE, Bossuyt PM, Boutron I, Hoffmann TC, Mulrow CD, et al. The PRISMA 2020 statement: an updated guideline for reporting systematic reviews. BMJ. 2021;372:n71. 10.1136/bmj.n71. 33782057 10.1136/bmj.n71 PMC8005924

[40] Ardern CL, Büttner F, Andrade R, Weir A, Ashe MC, Holden S, et al. Implementing the 27 PRISMA 2020 statement items for systematic reviews in the sport and exercise medicine, musculoskeletal rehabilitation and sports science fields: the PERSiST (implementing Prisma in Exercise, Rehabilitation, Sport medicine and SporTs science) guidance. Br J Sports Med. 2022;56:175–95. 10.1136/bjsports-2021-103987. 34625401 10.1136/bjsports-2021-103987 PMC8862073

[41] Van Alsenoy K, Van Der Linden ML, Girard O, Santos D. Increased footwear comfort is associated with improved running economy–a systematic review and meta-analysis. Eur J Sport Sci. 2021;23:121–33. 10.1080/17461391.2021.1998642. 34726119 10.1080/17461391.2021.1998642

[42] Ruiz-Alias SA, Molina-Molina A, Soto-Hermoso VM, García-Pinillos F. A systematic review of the effect of running shoes on running economy , performance and biomechanics: analysis by brand and model. Sports Biomech. 2022;22:388–409. 10.1080/14763141.2022.2089589. 35748066 10.1080/14763141.2022.2089589

[43] Munn Z, Stern C, Aromataris E, Lockwood C, Jordan Z. What kind of systematic review should I conduct? A proposed typology and guidance for systematic reviewers in the medical and health sciences. BMC Med Res Methodol. 2018;18:5. 10.1186/s12874-017-0468-4. 29316881 10.1186/s12874-017-0468-4 PMC5761190

[44] Sun X, Lam W, Zhang X, Wang J, Fu W. Systematic review of the role of footwear constructions in running biomechanics : implications for running-related injury and performance. J Sports Sci Med. 2020;19(1):20–37. 32132824 PMC7039038

[45] Sánchez-Ramírez C, Ramsey C, Palma-Oyarce V, Herrera-Hernández E, Aedo-Muñoz E. Heel-to-toe drop of running shoes: a systematic review of its biomechanical effects. Footwear Sci. 2023;15:77–101. 10.1080/19424280.2023.2180542.

[46] Sterne JAC, Savović J, Page MJ, Elbers RG, Blencowe NS, Boutron I, et al. RoB 2: A revised tool for assessing risk of bias in randomised trials. BMJ. 2019;366:l4898. 10.1136/bmj.l4898. 31462531 10.1136/bmj.l4898

[47] Knopp M, Muñiz-Pardos B, Wackerhage H, Schönfelder M, Guppy F, Pitsiladis Y, et al. Variability in running economy of Kenyan world-class and European amateur male runners with advanced footwear running technology: experimental and meta-analysis results. Sports Med. 2023;53:1255–71. 10.1007/s40279-023-01816-1. 36862339 10.1007/s40279-023-01816-1 PMC10185608

[48] Popay J, Roberts H, Sowden A, Petticrew M, Arai L, Rodgers M, et al. Guidance on the conduct of narrative synthesis in systematic reviews. A Product ESRC Methods Programme Version. 2006. 10.13140/2.1.1018.4643.

[49] Ryan R, Hill S. How to GRADE the quality of the evidence. Cochrane consumers and communication group. Cochrane. 2016;3. https://colorectal.cochrane.org/sites/colorectal.cochrane.org/files/uploads/how_to_grade.pdf. Accessed 18 Dec 2025.

[50] Higgins JPT, Green S. Cochrane handbook for systematic reviews of interventions. Chichester (UK): John Wiley & Sons. 2008. https://www.cochrane.org/authors/handbooks-and-manuals/handbook. Accessed 18 Dec 2025.

[51] Reinschmidt C, Nigg B. Influence of heel height on ankle joint moments in running. Med Sci Sports Exerc. 1995;27:410–6. 7752869

[52] Koegel J, Huerta S, Gambietz M, Ullrich M, Heyde C, Dorschky E, et al. Clustering runners’ response to different midsole stack heights: a field study. Sensors Basel. 2024;24:4694. 10.3390/s24144694. 39066091 10.3390/s24144694 PMC11280980

[53] Miyazaki T, Aimi T, Nakamura Y. Redistribution of knee and ankle joint work with different midsole thicknesses in non-rearfoot strikers during running: a cross-sectional study. Acta Bioeng Biomech. 2023;25:79–90. 10.37190/ABB-02202-2023-03. 38314579

[54] Barrons ZB, Wannop JW, Stefanyshyn DJ. The influence of midsole thickness on running turns. Footwear Sci. 2024;16:87–91. 10.1080/19424280.2024.2316345.

[55] TenBroek TM, Rodrigues PA, Frederick EC, Hamill J. Midsole thickness affects running patterns in habitual rearfoot strikers during a sustained run. J Appl Biomech. 2014;30:521–8. 10.1123/jab.2012-0224. 24615336 10.1123/jab.2012-0224

[56] TenBroek TM, Rodrigues P, Frederick EC, Hamill J. Effects of unknown footwear midsole thickness on running kinematics within the initial six minutes of running. Footwear Sci. 2013;5:27–37. 10.1080/19424280.2012.744360.

[57] Law MHC, Choi EMF, Law SHY, Chan SSC, Wong SMS, Ching ECK, et al. Effects of footwear midsole thickness on running biomechanics. J Sports Sci. 2019;37:1004–10. 10.1080/02640414.2018.1538066. 30358487 10.1080/02640414.2018.1538066

[58] Chambon N, Delattre N, Guéguen N, Berton E, Rao G. Is midsole thickness a key parameter for the running pattern? Gait Posture. 2014;40:58–63. 10.1016/j.gaitpost.2014.02.005. 24636223 10.1016/j.gaitpost.2014.02.005

[59] Barrons ZB, Wannop JW, Stefanyshyn DJ. The influence of footwear midsole thickness on running economy and frontal plane ankle stability. Footwear Sci. 2023;15:155–60. 10.1080/19424280.2023.2218321.

[60] Zhang Z, Lake M. A re-examination of the measurement of foot strike mechanics during running: the immediate effect of footwear midsole thickness. Front Sports Act Living. 2022;4:824183. 10.3389/fspor.2022.824183. 35557980 10.3389/fspor.2022.824183 PMC9086850

[61] Kettner C, Stetter B, Stein T. The effects of running shoe stack height on running style and stability during level running at different running speeds. Front Bioeng Biotechnol. 2025;13:1526752. 10.3389/fbioe.2025.1526752. 40059889 10.3389/fbioe.2025.1526752 PMC11885301

[62] Horvais N, Samozino P. Effect of midsole geometry on foot-strike pattern and running kinematics. Footwear Sci. 2013;5:81–9. 10.1080/19424280.2013.767863.

[63] Frank NS, Prentice SD, Callaghan JP. Local dynamic stability of the lower extremity in novice and trained runners while running in traditional and minimal footwear. Gait Posture. 2019;68:50–4. 10.1016/j.gaitpost.2018.10.034. 30458428 10.1016/j.gaitpost.2018.10.034

[64] Hannigan JJ, Pollard CD. Differences in running biomechanics between a maximal, traditional, and minimal running shoe. J Sci Med Sport. 2020;23:15–9. 10.1016/j.jsams.2019.08.008. 31501022 10.1016/j.jsams.2019.08.008

[65] Teng J, Qu F, Shen S, Jia SW, Lam WK. Effects of midsole thickness on ground reaction force, ankle stability, and sports performances in four basketball movements. Sports Biomech. 2022. 10.1080/14763141.2022.2112747. 36047733 10.1080/14763141.2022.2112747

[66] Kulmala JP, Kosonen J, Nurminen J, Avela J. Running in highly cushioned shoes increases leg stiffness and amplifies impact loading. Sci Rep. 2018;8:17496. 10.1038/s41598-018-35980-6. 30504822 10.1038/s41598-018-35980-6 PMC6269547

[67] Ernst M, Götze M, Müller R, Blickhan R. Vertical adaptation of the center of mass in human running on uneven ground. Hum Mov Sci. 2014;38:293–304. 10.1016/j.humov.2014.05.012. 25457426 10.1016/j.humov.2014.05.012

[68] Gholami M, Rezaei A, Cuthbert TJ, Napier C, Menon C. Lower body kinematics monitoring in running using fabric-based wearable sensors and deep convolutional neural networks. Sensors. 2019;19:5325. 10.3390/s19235325. 31816931 10.3390/s19235325 PMC6928687

[69] Trinler U, Schwameder H, Baker R, Alexander N. Muscle force estimation in clinical gait analysis using AnyBody and OpenSim. J Biomech. 2019;86:55–63. 10.1016/j.jbiomech.2019.01.045. 30739769 10.1016/j.jbiomech.2019.01.045

[70] Kainz H, Modenese L, Lloyd DG, Maine S, Walsh HPJ, Carty CP. Joint kinematic calculation based on clinical direct kinematic versus inverse kinematic gait models. J Biomech. 2016;49:1658–69. 10.1016/j.jbiomech.2016.03.052. 27139005 10.1016/j.jbiomech.2016.03.052

[71] Van Der Worp H, Vrielink JW, Bredeweg SW. Do runners who suffer injuries have higher vertical ground reaction forces than those who remain injury-free? A systematic review and meta-analysis. Br J Sports Med. 2016;50:450–7. 10.1136/bjsports-2015-094924. 26729857 10.1136/bjsports-2015-094924

[72] Schmida EA, Wille CM, Stiffler-Joachim MR, Kliethermes SA, Heiderscheit BC. Vertical loading rate is not associated with running injury, regardless of calculation method. Med Sci Sports Exerc. 2022;54:1382–8. 35320147 10.1249/MSS.0000000000002917 PMC9288487

[73] Whiting CS, Hoogkamer W, Kram R. Metabolic cost of level, uphill, and downhill running in highly cushioned shoes with carbon-fiber plates: graded running in modern marathon shoes. J Sport Health Sci. 2022;11:303–8. 10.1016/j.jshs.2021.10.004. 34740871 10.1016/j.jshs.2021.10.004 PMC9189710

[74] Barrons ZB, Wannop JW, Stefanyshyn DJ. The influence of footwear midsole thickness on running biomechanics and running economy in female and male runners. Footwear Sci. 2023;1(Suppl 1):S8-9. 10.1080/19424280.2023.2218321.

[75] Paquette MR, Melaro JA, Smith R, Moore IS. Time to stability of treadmill running kinematics in novel footwear with different midsole thickness. J Biomech. 2024;164:111984. 10.1016/j.jbiomech.2024.111984. 38330884 10.1016/j.jbiomech.2024.111984

[76] Bertschy M, Lino H, Healey L, Hoogkamer W. Effects of midsole stack height and foam on the metabolic cost of running. Footwear Sci. 2023;15(Suppl 1):S180–1. 10.1080/19424280.2023.2202942.

[77] Matias A, Outerleys J, Johnson C, Sacco ICN, Davis IS. Correlations between stack height differences in minimal shoes and impact loading. Footwear Sci. 2019;11(Suppl 1):S196–8. 10.1080/19424280.2019.1606330.

[78] Rodrigo-Carranza V, González-Mohíno F, Santos-Concejero J, González-Ravé JM. Influence of shoe mass on performance and running economy in trained runners. Front Physiol. 2020;11:573660. 10.3389/fphys.2020.573660. 33071828 10.3389/fphys.2020.573660 PMC7538857

[79] Rodrigo-Carranza V, Hoogkamer W, González-Ravé JM, Horta-Muñoz S, Serna-Moreno MdelC, Romero-Gutierrez A, et al. Influence of different midsole foam in advanced footwear technology use on running economy and biomechanics in trained runners. Scand J Med Sci Sports. 2024;34:1–10. 10.1111/sms.14526. 10.1111/sms.14526 37858294

[80] Kerdok AE, Biewener AA, Mcmahon TA, Weyand PG, Herr HM. Energetics and mechanics of human running on surfaces of different stiffnesses. J Appl Physiol. 2002;92:469–78. 10.1152/japplphysiol.01164.2000. 11796653 10.1152/japplphysiol.01164.2000

[81] Ferris DP, Liang K, Farley CT. Runners adjust leg stiffness for their first step on a new running surface. J Biomech. 1999;32:787–94. 10.1016/s0021-9290(99)00078-0. 10433420 10.1016/s0021-9290(99)00078-0

[82] Ferris DP, Louie M, Farley CT. Running in the real world adjusting leg stiffness for different surfaces. Proc Biol Sci. 265: 989–94. 10.1098/rspb.1998.0388 10.1098/rspb.1998.0388 PMC1689165 9675909

[83] Nilsson J, Thorstensson A. Ground reaction forces at different speeds of human walking and running. Acta Physiol Scand. 1989;136:217–27. 10.1111/j.1748-1716.1989.tb08655.x. 2782094 10.1111/j.1748-1716.1989.tb08655.x

[84] Schache AG, Blanch PD, Dorn TW, Brown NAT, Rosemond D, Pandy MG. Effect of running speed on lower limb joint kinetics. Med Sci Sports Exerc. 2011;43:1260–71. 10.1249/MSS.0b013e3182084929. 21131859 10.1249/MSS.0b013e3182084929

[85] Kimura A, Yokozawa T, Ozaki H. Clarifying the biomechanical concept of coordination through comparison with coordination in motor control. Front Sports Act Living. 2021;3:753062. 10.3389/fspor.2021.753062. 34723181 10.3389/fspor.2021.753062 PMC8551718

[86] Xu L, Wang Y, Wen X. The role of footwear in improving running economy: a systematic review with meta-analysis of controlled trials. Sci Rep. 2025;15:3963. 10.1038/s41598-025-88271-2. 39893208 10.1038/s41598-025-88271-2 PMC11787295

[87] Willer J, Allen SJ, Burden RJ, Folland JP. How humans run faster: the neuromechanical contributions of functional muscle groups to running at different speeds. Scand J Med Sci Sports. 2024. 10.1111/sms.14690. 39049546 10.1111/sms.14690

[88] Nüesch C, Roos E, Egloff C, Pagenstert G, Mündermann A. The effect of different running shoes on treadmill running mechanics and muscle activity assessed using statistical parametric mapping (SPM). Gait Posture. 2019;69:1–7. 10.1016/j.gaitpost.2019.01.013. 30658310 10.1016/j.gaitpost.2019.01.013

[89] Hamill J, Palmer C, Van Emmerik REA. Coordinative variability and overuse injury. Sports Med Arthrosc Rehabil Ther Technol. 2012;4:45. 10.1186/1758-2555-4-45. 23186012 10.1186/1758-2555-4-45 PMC3536567

